# Male and female Ethiopian and Kenyan runners are the fastest and the youngest in both half and full marathon

**DOI:** 10.1186/s40064-016-1915-0

**Published:** 2016-02-29

**Authors:** Beat Knechtle, Pantelis T. Nikolaidis, Vincent O. Onywera, Matthias A. Zingg, Thomas Rosemann, Christoph A. Rüst

**Affiliations:** Facharzt FMH für Allgemeinmedizin, Gesundheitszentrum St. Gallen, Vadianstrasse 26, St. Gallen, 9001 Switzerland; Institute of Primary Care, University of Zurich, Zurich, Switzerland; Department of Physical and Cultural Education, Hellenic Army Academy, Athens, Greece; Department of Recreation Management and Exercise Science, Kenyatta University, Nairobi, Kenya

**Keywords:** Age, Athletes, Endurance, Sex, Long-distance, Nationality, Running

## Abstract

In major marathon races such as the ‘World Marathon Majors’, female and male East African runners particularly from Ethiopia and Kenya are the fastest. However, whether this trend appears for female and male Ethiopians and Kenyans at recreational level runners (i.e. races at national level) and in shorter road races (e.g. in half-marathon races) has not been studied yet. Thus, the aim of the present study was to examine differences in the performance and the age of female and male runners from East Africa (i.e. Ethiopians and Kenyans) between half- and full marathons. Data from 508,108 athletes (125,894 female and 328,430 male half-marathoners and 10,205 female and 43,489 male marathoners) originating from 126 countries and competing between 1999 and 2014 in all road-based half-marathons and marathons held in one country (Switzerland) were analysed using Chi square (χ^2^) tests, mixed-effects regression analyses and one-way analyses of variance. In half-marathons, 48 women (0.038 %) and 63 men (0.019 %) were from Ethiopia and 80 women (0.063 %) and 134 men (0.040 %) from Kenya. In marathons, three women (0.029 %) and 15 men (0.034 %) were from Ethiopia and two women (0.019 %) and 33 men (0.075 %) from Kenya. There was no statistically significant association between the nationality of East Africans and the format of a race. In both women and men, the fastest race times in half-marathons and marathons were achieved by East African runners (p < 0.001). Ethiopian and Kenyan runners were the youngest in both sexes and formats of race (p < 0.001). In summary, women and men from Ethiopia and Kenya, despite they accounted for <0.1 % in half-marathons and marathons, achieved the fastest race times and were the youngest in both half-marathons and marathons. These findings confirmed in the case of half-marathon the trend previously observed in marathon races for a better performance and a younger age in East African runners from Ethiopia and Kenya.

## Background

Marathon and half-marathon races are very popular running events held all over the world with an increasing number of both races and participants during the last decades. For instance, in the USA, there were more than 1200 marathons held in 2014 compared to about 300 marathons held in 2000 (www.runningusa.org/2015-national-runner-survey). The number of successful marathon finishers increased from 25,000 in 1976 to the all-time high of 550,637 in 2014. Compared to marathons, however, most of the runners competed in the USA in half-marathons. The number of successful half-marathoners increased from 303,000 in 1990 to the all-time high of 2,046,600 in 2014 (www.runningusa.org/half-marathon-report-2015). In fact, 3.7 times more half-marathoners than marathoners competed in the USA in 2014. In smaller countries such as Switzerland in Europe, a total of 226,754 half-marathoners and 86,419 marathoners competed between 2000 and 2010 (Anthony et al. 2014). In other terms, 2.6 times more half-marathoners competed than marathoners. In 2010, 8690 women and 21,583 men finished a half-marathon in comparison to 2904 female and 9333 male finishers in 2000, respectively, corresponding to an increase of 299 % for women and of 231 % for men over 10 years. In contrast, the number of male and female full marathoners increased until 2005 only and decreased thereafter (Anthony et al. 2014).

The dominance of East-African women and men in marathon running is well known (Hamilton 2000; Onywera et al. 2006; Tucker et al. 2015; Wilber and Pitsiladis 2012). Athletes from both Ethiopia and Kenya dominate marathon running for a long time (www.iaaf.org). In the top list of the International Association of Athletics Federations (IAAF) for male marathoners, the first best 37 marathon race times were achieved by athletes from Ethiopia and Kenya (www.iaaf.org/records/toplists/road-running/marathon/outdoor/men/senior). In women, however, the three fastest marathon race times were achieved by an athlete from Great Britain followed by two female marathoners from Kenya (www.iaaf.org/records/toplists/road-running/marathon/outdoor/women/senior). In the ‘World Marathon Majors’ with the largest city marathons worldwide, female and male champions are exclusively from East African particularly from Ethiopia and Kenya (www.worldmarathonmajors.com/champions/current-champions).

The reasons for the dominance of East-African runners in long and middle distance running events such as marathons included environmental conditions such as a specific geographic background (Onywera et al. 2006; Scott et al. 2003; Tucker et al. 2015). The dominance of East-African distance runners is primarily a Kenyan phenomenon, with majority of the Kenyan runners originating from the Kalenjin tribe in general and the Nandi sub-tribe in particular (Onywera et al. 2006; Tucker et al. 2015). Similar to Kenyan runners, elite Ethiopian runners are also of a distinct environmental background where marathoners mainly originate from the altitudinous regions of Arsi and Shewa (Scott et al. 2003).

However, there is paucity of information with regards to basic characteristics such as age and trends in performance of East-African half-marathoners (Aschmann et al. 2013; Cribari et al. 2013). These studies investigated all African half- and full marathoners competing in one country (Switzerland) together without a separation of East-African runners in their nationalities (Aschmann et al. 2013) or investigated a limited sample of the best athletes (Cribari et al. 2013). Indeed, East African runners particularly those from Ethiopia and Kenya account for the largest percentage of African runners in half-marathon and marathon (Aschmann et al. 2013). A recent study showed different barriers across both sex and distance (Wegner et al. 2015); hence, these trends might vary between half-marathon and marathon. The knowledge of East African’s basic characteristics such as age, participation and performance trends might help coaches, fitness trainers and sports scientists to improve their understanding of half-marathon’s demands.

Therefore, the aim of this study was to investigate performance and age of Ethiopian and Kenyan half- and full marathoners who competed between 1999 and 2014 in races held within one country (Switzerland) in a sample of more than 500,000 successful finishers. We hypothesized that female and male runners from Ethiopia and Kenya would also be the fastest in half-marathon races.

## Methods

### Ethics

The study was approved by the Institutional Review Board of St. Gallen, Switzerland, with waiver of the requirement for informed consent given that the study involved the analysis of publicly available data.

### Data collection and data analysis

All half-marathons and marathons held in Switzerland from 1999 to 2014 were identified by using ‘Laufkalender Schweiz’ (www.laufkalender.ch). Since 1999, all running races in Switzerland started with an electronic chip system and full race results (i.e. name, age, sex, nationality and race time of the finishers) were available since then on the website of the specific races. Of all races, only those half-marathons and marathons were considered which were held on a road, not on a trail. No mountain marathons were included; start and finish of the race had to be on the same altitude. Athletes with missing age and/or missing nationality were excluded from data analysis. In order to avoid a selection bias due to a limitation to top runners, we considered all finishers from all countries. To investigate a trend in participation and performance, athletes from countries where at least one women and/or one man competed in at least 8 years (i.e. half of the investigated period of time) were considered.

### Statistical analysis

Each set of data was tested for normal distribution (D’Agostino and Pearson omnibus normality test) and for homogeneity of variances (Levine’s test) prior to statistical analyses. Trends in participation across calendar years were analysed using regression analysis with linear growth equation models. Differences in the participation of East African runners by nationality and sex to half-marathons and marathon were examined by using Chi square (χ^2^) test. To investigate changes in performance across calendar years, we used a mixed-effects regression model with running speed as the dependent variable. We analysed women and men separately for each country for both half-marathon and marathon and included calendar year, sex, centered age, and squared centered age as fixed variables. To investigate changes in age across calendar years, we used a mixed-effects regression model with age as the dependent variable. For the change in age over time, we combined women and men for each country and included sex and calendar year as fixed variables. Differences in age and performance between athletes from multiple countries were compared using one-way analysis of variance (ANOVA) with subsequent Tukey’s multiple comparison tests with a single pooled variance. Statistical analyses were performed using IBM SPSS Statistics (Version 22, IBM SPSS, Chicago, IL, USA) and GraphPad Prism (Version 6.01, GraphPad Software, La Jolla, CA, USA). Significance was accepted at p < 0.05 (two-sided for *t* tests). Data in the text and tables are given as mean ± standard deviation (SD).

## Results

### Participation

Data from a total of 508,108 (125,894 female and 328,430 male half-marathoners and 10,205 female and 43,489 male marathoners) athletes was considered. These runners originated from a total of 126 countries spread around the globe. Table 1 summarizes the athletes from the considered countries for data analysis across calendar years in half-marathons (35 countries) and marathons (15 countries).

**Table 1:** Number of women and men considered by nationality for half-marathons and marathons, sorted by the overall participation

Country	Number of years	Number of women	Number of men	Overall
Half-marathon				
Ethiopia	14	24	48	72
Kenya	14	80	134	214
Switzerland	15	108,509	283,353	391,862
Germany	15	5782	16,332	22,114
France	15	5889	14,511	20,400
Italy	15	984	2820	3804
Austria	15	897	2141	3038
Great Britain	15	872	2124	2996
USA	15	331	909	1240
Liechtenstein	15	304	675	979
Belgium	14	180	567	747
Spain	15	227	483	710
Canada	15	208	411	619
Netherlands	15	163	438	601
Japan	15	167	398	565
Sweden	14	111	246	357
Finland	13	85	223	308
Poland	14	100	199	299
Portugal	15	56	190	246
Denmark	15	60	186	246
Luxembourg	15	85	146	231
Hungary	14	51	175	226
Czech Republic	15	62	162	224
Australia	14	48	139	187
Russia	14	62	109	171
Norway	15	55	110	165
Brazil	10	40	86	126
Mexico	10	28	71	99
Greece	12	20	74	94
Republic of South Africa	11	32	44	76
Israel	8	12	57	69
India	8	23	45	68
Ireland	14	11	24	35
Argentina	8	13	22	35
Slovenia	8	7	20	27
Marathon				
Ethiopia	8	3	15	18
Kenya	13	2	33	35
Switzerland	15	8376	35,084	43,460
Germany	15	683	3319	4002
France	15	539	2428	2967
Austria	15	119	375	494
Great Britain	15	97	389	486
Italy	15	67	357	424
USA	11	40	268	308
Japan	15	48	119	167
Belgium	8	14	123	137
Canada	12	30	103	133
Liechtenstein	11	25	78	103
Spain	8	18	57	75
Poland	8	14	52	66

In half-marathons, 48 women (0.038 %) and 63 men (0.019 %) originated from Ethiopia and 80 women (0.063 %) and 134 men (0.040 %) from Kenya. In marathons, three women (0.029 %) and 15 men (0.034 %) were from Ethiopia and two women (0.019 %) and 33 men (0.075 %) from Kenya. There was no statistically significant association between the nationality of East Africans and the format of the race [χ^2^(1) = 0.001, p = 0.978]; that was, both Ethiopians and Kenyans equally participated to half-marathons versus marathons. Also, there was no association between male East Africans and the format of the race [χ^2^(1) = 0.001, p = 0.922]; i.e. both male Ethiopians and Kenyans accounted equally to the two formats.

Most of the successful finishers originated from Switzerland, Germany and France in both half-marathons and marathons. In half-marathons, the number of women (r^2^ = 0.98, p < 0.0001) and men (r^2^ = 0.98, p < 0.0001) increased significantly. Similarly, the number of women (r^2^ = 0.46, p = 0.0041) and men (r^2^ = 0.51, p = 0.0019) increased significantly in marathons. Regarding the considered countries, the number of female half-marathoners from Canada (r^2^ = 0.81, p = 0.002), Germany (r^2^ = 0.97, p = 0.005), Switzerland (r^2^ = 0.97, p = 0.005) and Belgium (r^2^ = 0.72, p < 0.0001) increased significantly. For male half-marathoners, the number of participants from France (r^2^ = 0.97, p = 0.018), Great Britain (r^2^ = 0.88, p = 0.036), Principality of Liechtenstein (r^2^ = 0.87, p < 0.0001), Poland (r^2^ = 0.65, p < 0.0001), South Africa (r^2^ = 0.63, p = 0.006) and Argentina (r^2^ = 0.70, p < 0.0001) increased significantly. In marathoners, there was no significant increase in the number of men regarding the country. In women, however, participants from France (r^2^ = 0.46, p = 0.0275) and Japan (r^2^ = 0.47, p = 0.0039) increased significantly their numbers.

### Trends in performance and age across calendar years

Table 2 shows the running speed of the female and male half-marathoners. Running speed decreased significantly in women from France, Switzerland, and Australia, but increased in women from Norway and Portugal (Table 3). In men, running speed decreased in athletes from Germany (Table 4). Table 5 presents running speed of female and male marathoners. Running speed remained unchanged in female marathoners (Table 6) but increased in British men (Table 7). Table 8 presents the age of the female and male half-marathoners. Age increased significantly across calendar years in women from Austria and Norway and in men from Japan and Norway (Table 9). In marathoners (Table 10), age decreased significantly in men from Italy and Principality of Liechtenstein, but increased significantly in men from Poland (Table 11).

**Table 2:** Running speed (km/h) with mean ± SD for the annual fastest female and male East-African and Non-African half-marathoners

	1999	2000	2001	2002	2003	2004	2005	2006
Women								
Ethiopia	8.60	14.66 ± 7.42	8.10		19.57	19.59	19.63	14.14 ± 5.27
Kenya		14.79 ± 7.29		9.43 ± 0.35	14.99 ± 8.04	14.18 ± 4.69	16.52 ± 3.36	18.84 ± 1.89
Austria	7.49 ± 3.04	8.02 ± 3.64	7.84 ± 3.30	8.02 ± 3.06	7.98 ± 2.98	8.78 ± 3.59	8.44 ± 3.43	8.99 ± 3.54
Canada		5.07 ± 1.30	7.78 ± 4.39	7.16 ± 3.70	5.86 ± 2.10	9.13 ± 4.75	7.76 ± 2.72	7.01 ± 2.56
Czech Republic	5.09 ± 1.34	4.51	8.13 ± 4.82	5.63 ± 0.79	8.85 ± 3.83	10.83 ± 2.99	8.11 ± 3.16	10.13
Denmark		10.81 ± 0.83	7.23 ± 4.15	8.84 ± 4.10	10.42 ± 1.16	9.58 ± 3.40	7.49 ± 2.77	6.87 ± 2.85
Spain	9.87 ± 3.23	10.25 ± 2.32	10.53 ± 4.01	10.02 ± 3.15	11.01 ± 1.86	9.78 ± 2.81	10.07 ± 3.05	10.69 ± 2.29
France	10.21 ± 3.23	9.99 ± 3.02	9.47 ± 3.25	9.94 ± 3.28	9.72 ± 3.17	9.68 ± 3.40	9.70 ± 3.48	9.27 ± 3.42
Great Britain	9.81 ± 1.99	9.83 ± 2.84	9.18 ± 3.29	8.33 ± 3.13	10.38 ± 3.03	9.63 ± 3.20	10.26 ± 2.93	9.38 ± 3.08
Germany	8.49 ± 2.97	8.35 ± 3.47	8.46 ± 3.15	8.39 ± 3.22	8.61 ± 3.35	8.31 ± 3.27	8.48 ± 3.40	8.31 ± 3.17
Italy	9.76 ± 3.07	10.02 ± 3.26	10.4 ± 3.10	11.7 ± 2.62	10.01 ± 3.31	11.35 ± 2.92	10.68 ± 3.10	10.98 ± 3.11
Japan	6.21 ± 2.80	6.15 ± 2.47	7.35 ± 2.53	7.94 ± 2.95	6.57 ± 3.37	6.20 ± 2.48	6.52 ± 1.96	8.09 ± 2.77
Liechtenstein	10.21 ± 2.09	10.17 ± 2.32	11.49 ± 2.51	11.51 ± 2.25	10.96 ± 2.63	10.85 ± 3.55	11.26 ± 1.50	9.94 ± 2.66
Luxembourg	5.87 ± 1.58	7.80 ± 3.15	8.15 ± 4.64	8.19 ± 3.56	8.25 ± 3.33	6.86 ± 2.39	7.45 ± 1.22	8.61 ± 3.06
Netherlands	10.90 ± 1.25	11.56 ± 2.30	10.79 ± 1.16	11.47 ± 2.19	9.51 ± 2.63	10.12 ± 2.68	10.45 ± 4.47	8.97 ± 3.29
Norway		6.83 ± 3.05	7.12 ± 4.66	9.7 ± 1.93	4.56	9.59 ± 3.81	10.27 ± 3.84	8.45 ± 4.13
Portugal		10.97	13.53	8.41 ± 3.32	11.08 ± 2.74	10.12 ± 3.31	10.15 ± 3.98	11.52 ± 3.36
Switzerland	10.59 ± 2.97	10.75 ± 2.87	10.63 ± 2.91	10.63 ± 2.92	10.58 ± 2.90	10.57 ± 2.90	10.45 ± 2.93	10.51 ± 2.90
USA	12.05 ± 0.61	9.50 ± 2.92	9.39 ± 3.02	8.67 ± 3.46	7.90 ± 3.10	8.58 ± 3.60	8.34 ± 3.27	8.88 ± 2.62
Australia		10.96	9.08 ± 4.03	9.89 ± 3.60	9.85 ± 4.61		8.09 ± 3.28	8.12 ± 3.14
Belgium	8.22 ± 3.49		9.62 ± 5.59	7.70 ± 2.50	9.50 ± 1.87	7.88 ± 2.19	8.99 ± 3.28	7.83 ± 3.10
Hungary	8.44	9.09		8.46	11.79	10.96	11.62	10.07 ± 3.41
Ireland	9.44 ± 4.42	11.13	10.45 ± 0.33	8.60 ± 3.12	11.12 ± 3.02	10.49 ± 2.69		11.99 ± 1.66
Poland	8.19 ± 5.46		5.17 ± 1.00	4.39	9.58 ± 3.89	7.41 ± 2.78	7.49 ± 2.98	7.80 ± 3.18
Russia	11.19	9.73 ± 0.24	9.60	8.00		9.05 ± 3.13	7.31 ± 2.71	8.42 ± 2.99
Sweden	5.04		7.05 ± 3.13	11.66 ± 1.29	9.58 ± 3.82	10.02 ± 2.09	9.35 ± 3.67	7.47 ± 2.66
Finland	10.80 ± 0.50	5.07		8.42 ± 4.75		6.76 ± 3.14	6.14 ± 2.01	7.43 ± 0.33
Greece					5.82	6.39	10.65	12.02
South Africa				6.76 ± 2.45	11.59	12.73	5.32	5.06 ± 0.12
Brazil	9.95 ± 0.67	12.10 ± 2.16		5.49	10.28	11.06	8.72 ± 4.37	
Mexico						9.76		10.07 ± 1.18
Argentina				9.74				10.44
India		9.74				10.48	9.63 ± 1.48	10.62 ± 1.09
Israel						4.37	4.50	
Slovenia							5.06	
Men								
Ethiopia	9.71 ± 2.04	19.11	13.41 ± 6.70	8.13 ± 0.79	12.93 ± 7.40	10.84 ± 5.18	8.39 ± 0.39	12.24 ± 5.34
Kenya	12.76 ± 4.68	14.62 ± 5.32	12.24 ± 5.38	14.7 ± 5.13	12.31 ± 6.99	10.78 ± 4.83	15.35 ± 5.68	11.47 ± 4.39
Austria		9.36 ± 3.57	11.77 ± 2.60	9.87 ± 3.42	8.97 ± 2.26	11.03 ± 1.91	8.98 ± 3.12	7.38 ± 2.43
Canada	7.81 ± 3.15	6.99 ± 2.69	8.32 ± 3.28	7.78 ± 3.05	8.95 ± 3.65	7.29 ± 2.87	6.35 ± 2.61	5.91 ± 2.34
Czech Republic	8.06 ± 2.00	10.45 ± 3.87	10.3 ± 2.72	11.15 ± 3.54	8.85 ± 4.47	10.01 ± 2.75	9.63 ± 3.10	8.94 ± 2.96
Denmark		6.68 ± 1.70	7.04 ± 2.49	8.65 ± 2.96	6.91 ± 2.03	8.25 ± 2.94	8.65 ± 2.90	9.15 ± 3.74
Spain	10.63 ± 3.75	11.23 ± 1.76	9.84 ± 3.25	8.32 ± 3.01	9.24 ± 2.84	8.88 ± 3.00	8.41 ± 3.41	9.07 ± 2.89
France	9.82 ± 3.37	9.43 ± 3.35	9.47 ± 3.33	9.72 ± 3.37	9.49 ± 3.29	9.39 ± 3.32	9.56 ± 3.35	9.83 ± 3.33
Great Britain	9.49 ± 2.92	9.32 ± 3.16	9.31 ± 3.16	9.34 ± 3.09	9.89 ± 2.84	9.06 ± 3.04	9.19 ± 3.16	9.37 ± 3.11
Germany	8.58 ± 3.26	8.56 ± 3.25	8.42 ± 3.16	8.58 ± 3.28	8.35 ± 3.23	8.44 ± 3.21	8.28 ± 3.23	8.60 ± 3.18
Italy	10.55 ± 3.16	10.55 ± 3.01	10.73 ± 3.05	10.54 ± 2.86	10.59 ± 3.17	10.64 ± 3.23	10.82 ± 3.00	10.68 ± 3.18
Japan	4.14 ± 0.31	6.47 ± 3.61	6.51 ± 3.43	5.55 ± 2.22	7.03 ± 2.61	7.03 ± 3.97	7.03 ± 3.03	5.93 ± 2.90
Liechtenstein	10.92 ± 2.10	10.09 ± 2.76	10.61 ± 2.48	10.57 ± 2.78	11.17 ± 1.84	10.76 ± 2.48	10.57 ± 3.07	10.84 ± 2.37
Luxembourg	9.41 ± 3.96	7.14 ± 3.04	6.82 ± 2.23	6.88 ± 2.58	6.85 ± 2.40	8.23 ± 2.93	8.04 ± 3.00	7.48 ± 3.02
Netherlands	10.39 ± 4.64	9.52 ± 2.15	10.99 ± 3.41	9.02 ± 3.67	8.85 ± 3.23	9.75 ± 3.09	9.02 ± 3.05	10.63 ± 4.05
Norway	12.91	7.29 ± 0.30	10.91 ± 2.32	9.11 ± 2.42	8.20 ± 3.73	9.20 ± 2.66	9.96 ± 1.05	9.71 ± 3.80
Portugal	12.04 ± 1.87	12.87 ± 0.62	12.37 ± 2.79	11.63 ± 3.25	9.60 ± 2.92	11.82 ± 1.97	9.39 ± 3.48	11.76 ± 2.08
Switzerland	10.31 ± 3.05	10.52 ± 2.96	10.50 ± 2.94	10.38 ± 2.96	10.43 ± 2.98	10.43 ± 2.95	10.41 ± 2.98	10.44 ± 2.99
USA	7.44 ± 3.01	7.83 ± 3.35	7.44 ± 3.07	7.79 ± 3.31	8.02 ± 2.75	8.25 ± 2.69	8.97 ± 2.72	8.41 ± 3.20
Australia		9.36 ± 3.57	11.77 ± 2.60	9.87 ± 3.42	8.97 ± 2.26	11.03 ± 2.91	8.98 ± 3.12	7.38 ± 2.43
Belgium	9.57 ± 3.14	9.7 ± 2.92	9.93 ± 3.16	8.68 ± 3.45	9.00 ± 3.16	9.54 ± 3.40	9.93 ± 2.43	9.17 ± 3.37
Hungary		12.34	11.70 ± 1.26	8.79 ± 3.02	10.93 ± 3.01	9.21 ± 2.83	11.46 ± 2.42	11.23 ± 2.74
Ireland	9.17 ± 5.65	9.84 ± 2.78	7.95 ± 2.65	7.14 ± 3.44	8.15 ± 3.25	7.55 ± 3.84	8.57 ± 3.16	8.48 ± 3.58
Poland	6.20 ± 1.79	5.52 ± 0.84	8.77 ± 2.71	8.69 ± 5.06	11.68 ± 3.38	9.43 ± 5.84	9.28 ± 2.95	8.93 ± 2.87
Russia		8.17 ± 4.61	8.53 ± 3.25	9.29 ± 2.78	7.45 ± 2.53	8.18 ± 2.56	8.61 ± 2.09	9.26 ± 2.81
Sweden	8.29 ± 4.64	7.26 ± 4.06	9.08 ± 2.67	7.36 ± 3.64	7.98 ± 3.01	8.33 ± 3.71	7.95 ± 3.48	8.04 ± 3.23
Finland	7.66 ± 4.62	7.36 ± 2.81	7.37 ± 2.67	6.95 ± 2.59	5.22 ± 1.75	7.56 ± 2.68	8.10 ± 2.65	8.24 ± 3.08
Greece	5.65	5.60	6.52 ± 0.82	6.52 ± 3.20	11.17 ± 1.66	9.93 ± 4.58	8.53 ± 3.53	8.40 ± 4.46
South Africa	4.24			4.80		7.93 ± 3.70	10.72	7.91 ± 4.32
Brazil	5.21 ± 0.56	7.45 ± 3.02	8.91 ± 3.46		6.97 ± 3.01			6.70 ± 1.90
Mexico	10.67	10.56	6.32	7.00 ± 2.84	8.26 ± 3.64	6.28 ± 2.79	5.68	11.90 ± 0.42
Argentina					9.24	6.13	4.24	5.96
India		9.57 ± 2.06	9.86 ± 0.45	7.89	9.92 ± 0.88		9.93 ± 0.78	9.58 ± 0.88
Israel		10.44 ± 3.74		6.05 ± 0.69	13.25 ± 0.04	7.88 ± 4.88	5.88	6.13 ± 1.58
Slovenia		5.75	6.89	5.58 ± 1.53	11.48	11.62	8.49 ± 5.25	8.89 ± 3.77

	2007	2008	2009	2010	2011	2012	2013	2014
Women								
Ethiopia	9.90 ± 3.96	15.01 ± 4.45	12.86 ± 5.72	13.86 ± 5.98	11.05 ± 4.50	15.73 ± 5.65	10.44 ± 5.79	12.03 ± 4.74
Kenya	13.44 ± 4.18	16.39 ± 4.74	16.66 ± 5.50	13.39 ± 7.36	11.63 ± 4.81	14.07 ± 5.28	15.96 ± 5.42	12.08 ± 5.73
Austria	7.71 ± 2.64	7.63 ± 2.90	7.56 ± 2.55	8.10 ± 3.15	7.79 ± 3.13	8.27 ± 3.31	7.82 ± 3.26	7.84 ± 3.14
Canada	6.09 ± 3.02	7.10 ± 3.19	7.88 ± 3.84	7.86 ± 4.29	7.45 ± 3.47	7.14 ± 3.04	7.13 ± 2.98	7.59 ± 3.34
Czech Republic	7.09 ± 2.73	12.49 ± 1.72	9.53 ± 4.79	9.46 ± 7.30	9.44 ± 3.61	8.54 ± 3.36	14.24 ± 5.94	10.53 ± 2.73
Denmark	7.86 ± 3.89	5.19	6.82 ± 2.96	9.61 ± 2.48	7.86 ± 2.83	9.65 ± 3.71	8.04 ± 3.40	10.24 ± 3.50
Spain	9.87 ± 3.40	9.55 ± 3.32	9.55 ± 2.86	11.20 ± 2.95	9.26 ± 3.37	10.49 ± 2.88	9.54 ± 3.55	9.56 ± 3.45
France	9.35 ± 3.40	9.82 ± 3.32	9.58 ± 3.37	9.45 ± 3.31	9.44 ± 3.32	9.54 ± 3.17	9.40 ± 3.26	9.28 ± 3.31
Great Britain	8.35 ± 3.58	9.53 ± 2.97	9.48 ± 3.21	9.63 ± 3.01	9.03 ± 3.13	8.79 ± 3.13	8.73 ± 3.00	9.14 ± 3.28
Germany	8.58 ± 3.24	8.43 ± 3.09	8.61 ± 3.23	8.39 ± 3.08	8.31 ± 3.13	8.50 ± 3.19	8.16 ± 3.27	8.42 ± 3.21
Italy	10.91 ± 3.34	11.17 ± 2.97	11.30 ± 3.06	10.91 ± 3.55	10.82 ± 3.13	10.00 ± 3.40	10.74 ± 3.07	10.47 ± 2.92
Japan	5.93 ± 2.94	6.03 ± 2.53	5.64 ± 1.97	6.55 ± 2.41	5.72 ± 2.53	5.41 ± 1.78	5.55 ± 2.90	6.95 ± 3.00
Liechtenstein	11.17 ± 1.82	10.81 ± 1.91	11.11 ± 2.31	10.36 ± 2.96	11.28 ± 2.94	11.16 ± 2.53	10.63 ± 2.92	11.33 ± 2.54
Luxembourg	8.11 ± 2.34	8.14 ± 3.27	9.20 ± 2.78	8.36 ± 2.96	10.69 ± 2.62	8.00 ± 3.03	7.80 ± 3.28	7.23 ± 2.42
Netherlands	9.01 ± 3.60	9.18 ± 3.27	8.96 ± 3.39	8.96 ± 3.48	9.19 ± 3.50	9.45 ± 3.83	9.54 ± 3.35	9.85 ± 3.10
Norway	9.03 ± 3.29	7.62 ± 3.02	8.66 ± 0.69	11.22 ± 5.16	10.88 ± 1.43	12.37 ± 0.60	11.79 ± 1.32	11.12 ± 2.75
Portugal	10.08 ± 3.62	13.55 ± 5.79	13.38 ± 1.35	12.12 ± 0.72	12.47	15.11 ± 3.09	10.49 ± 4.48	12.28 ± 2.50
Switzerland	10.35 ± 2.97	10.48 ± 2.92	10.55 ± 2.90	10.50 ± 2.92	10.53 ± 2.94	10.46 ± 2.94	10.43 ± 2.95	10.41 ± 2.95
USA	8.80 ± 3.00	8.81 ± 3.73	9.07 ± 3.17	8.69 ± 3.81	8.54 ± 2.97	9.19 ± 3.06	8.78 ± 2.88	8.20 ± 3.29
Australia	8.24 ± 3.18	8.71	10.99 ± 0.60	7.37 ± 3.14	10.75 ± 2.72	6.52 ± 1.65	6.99 ± 3.22	7.60 ± 2.60
Belgium	7.98 ± 3.11	7.27 ± 2.86	8.29 ± 3.30	8.01 ± 3.48	8.66 ± 3.23	9.60 ± 3.07	8.23 ± 3.12	9.32 ± 3.10
Hungary	9.16 ± 1.44	11.03	10.16 ± 2.24	11.68	9.79 ± 3.84	12.71 ± 2.62	11.64 ± 1.45	10.94 ± 2.68
Ireland	10.68	10.91	9.53 ± 2.66	9.55 ± 4.34	8.61 ± 3.35	9.73 ± 2.70	10.25 ± 3.59	8.47 ± 3.34
Poland	7.86 ± 3.43	10.36 ± 3.87	8.64 ± 2.75	8.07 ± 4.13	11.18 ± 3.66	7.73 ± 5.63	10.67 ± 3.45	8.29 ± 2.65
Russia	6.06	8.62 ± 1.93	11.04 ± 4.56	11.40 ± 2.66	10.26 ± 2.10	8.33 ± 2.18	9.97 ± 3.41	10.18 ± 0.66
Sweden	7.31 ± 3.34	7.16 ± 2.98	9.39 ± 3.78	9.73 ± 3.90	7.98 ± 2.84	7.57 ± 2.82	7.36 ± 3.03	8.42 ± 3.20
Finland	8.74 ± 6.63	5.40 ± 1.36	6.50 ± 3.09	5.54 ± 1.03	5.97 ± 2.95	6.18 ± 1.90	5.90 ± 1.85	8.03 ± 3.53
Greece	10.02 ± 1.19	7.17	5.08	9.06	6.80	7.71 ± 3.94	11.79 ± 1.20	9.14 ± 2.16
South Africa	11.31 ± 1.03	8.83 ± 2.10	8.22 ± 2.89		9.44 ± 1.14	9.04 ± 3.87	7.47 ± 3.19	7.43
Brazil	9.84 ± 2.47	9.45 ± 2.48	9.95 ± 1.26	6.80 ± 3.46	7.80 ± 3.30	10.23 ± 1.14	9.27 ± 1.28	8.98 ± 2.97
Mexico	10.97 ± 0.92	9.24 ± 0.15	11.97 ± 2.03	7.75 ± 2.36	6.81 ± 2.78	7.82 ± 3.91	7.45	9.25 ± 1.79
Argentina	12.68		11.85	4.50 ± 0.25	4.98	10.69 ± 1.09	12.53 ± 2.37	10.44 ± 1.89
India	9.85 ± 0.91		9.09		10.01 ± 0.93		10.92 ± 1.75	10.8 ± 1.75
Israel			13.38	9.64	5.41 ± 0.18	8.74	8.56 ± 3.24	13.18 ± 1.01
Slovenia	5.61		9.28 ± 4.70		7.82 ± 3.48			5.54
Men								
Ethiopia	10.80 ± 4.09	15.38 ± 5.10	9.87 ± 4.18	8.75 ± 0.70	11.34 ± 4.62	7.94 ± 0.52	13.60 ± 4.71	10.78 ± 4.94
Kenya	14.85 ± 5.54	12.67 ± 5.10	13.56 ± 4.77	11.77 ± 4.17	11.58 ± 4.70	10.86 ± 4.81	10.42 ± 3.17	13.14 ± 4.80
Austria	9.36 ± 3.01	10.55 ± 2.78	11.75 ± 2.30	9.81 ± 2.90	8.21 ± 2.92	9.43 ± 3.00	8.31 ± 3.18	9.25 ± 2.82
Canada	6.79 ± 3.43	6.33 ± 3.14	7.63 ± 3.17	7.77 ± 3.07	7.24 ± 2.77	7.5 ± 3.20	7.62 ± 3.15	8.29 ± 3.34
Czech Republic	8.35 ± 3.03	8.47 ± 3.99	8.23 ± 4.09	9.64 ± 3.40	6.25 ± 1.60	7.84 ± 2.92	7.51 ± 2.93	9.10 ± 3.82
Denmark	9.08 ± 2.97	9.36 ± 2.55	8.59 ± 3.08	7.92 ± 2.65	5.99 ± 2.49	7.08 ± 2.70	8.41 ± 3.14	8.02 ± 2.96
Spain	9.76 ± 2.91	10.74 ± 2.85	10.30 ± 2.59	8.74 ± 2.87	8.61 ± 3.40	10.22 ± 2.92	9.76 ± 3.37	8.79 ± 3.03
France	9.56 ± 3.34	9.48 ± 3.26	9.46 ± 3.33	9.52 ± 3.33	9.43 ± 3.35	9.38 ± 3.30	9.50 ± 3.34	9.54 ± 3.30
Great Britain	9.29 ± 2.94	8.88 ± 3.06	8.51 ± 3.06	8.88 ± 3.16	9.00 ± 3.12	8.95 ± 3.29	8.63 ± 3.24	8.66 ± 3.16
Germany	8.75 ± 3.26	8.57 ± 3.25	8.79 ± 3.32	8.34 ± 3.13	8.35 ± 3.17	8.34 ± 3.24	8.39 ± 3.15	8.29 ± 3.17
Italy	10.56 ± 3.32	10.90 ± 3.04	10.54 ± 3.27	10.41 ± 3.14	10.24 ± 3.32	10.10 ± 3.27	10.09 ± 3.28	10.18 ± 3.37
Japan	6.95 ± 3.17	5.64 ± 2.41	6.15 ± 2.52	6.94 ± 3.02	7.00 ± 3.13	7.32 ± 3.23	6.37 ± 2.84	6.20 ± 2.44
Liechtenstein	10.65 ± 3.20	10.17 ± 2.68	10.84 ± 2.43	10.83 ± 2.81	10.86 ± 2.80	10.41 ± 2.80	10.23 ± 2.89	10.18 ± 2.81
Luxembourg	9.47 ± 3.24	8.76 ± 3.63	7.81 ± 2.80	8.34 ± 2.30	8.43 ± 2.69	7.60 ± 2.54	6.68 ± 2.23	8.15 ± 3.89
Netherlands	9.21 ± 3.14	9.34 ± 3.31	9.71 ± 3.49	9.27 ± 3.03	10.31 ± 3.35	8.90 ± 3.29	9.65 ± 3.06	8.94 ± 3.51
Norway	8.12 ± 2.09	8.86 ± 0.28	10.53 ± 2.96	7.19 ± 3.29	7.84 ± 2.91	9.80 ± 3.54	8.92 ± 3.32	10.12 ± 3.20
Portugal	11.48 ± 2.40	10.45 ± 2.55	11.52 ± 2.59	10.86 ± 3.65	11.52 ± 4.09	11.34 ± 1.68	9.84 ± 3.66	10.65 ± 2.92
Switzerland	10.43 ± 2.99	10.46 ± 2.96	10.45 ± 2.95	10.41 ± 2.98	10.43 ± 2.96	10.38 ± 2.99	10.39 ± 3.02	10.44 ± 2.99
USA	8.41 ± 2.91	8.05 ± 2.93	7.87 ± 2.89	8.65 ± 2.76	8.38 ± 3.18	8.01 ± 3.03	7.74 ± 3.15	8.08 ± 3.16
Australia	9.36 ± 3.01	10.55 ± 2.78	11.75 ± 2.30	9.81 ± 2.90	8.21 ± 2.92	9.43 ± 3.00	8.31 ± 3.18	9.25 ± 2.82
Belgium	9.49 ± 2.92	8.46 ± 3.22	9.20 ± 2.78	8.41 ± 3.00	8.43 ± 3.07	7.31 ± 2.52	8.33 ± 2.85	8.86 ± 2.86
Hungary	8.52 ± 3.40	9.11 ± 4.00	9.54 ± 2.78	9.24 ± 2.79	9.74 ± 2.76	9.55 ± 3.17	11.73 ± 1.69	10.21 ± 2.69
Ireland	8.90 ± 2.50	9.31 ± 2.85	8.80 ± 3.29	10.25 ± 3.17	9.36 ± 3.08	8.56 ± 2.93	7.67 ± 2.97	8.59 ± 3.40
Poland	7.48 ± 3.00	9.23 ± 3.36	9.01 ± 4.29	8.18 ± 3.84	7.52 ± 2.96	8.20 ± 3.84	8.28 ± 3.32	9.37 ± 3.35
Russia	11.05 ± 2.22	8.55 ± 3.57	7.65 ± 3.03	8.00 ± 2.78	7.89 ± 2.98	8.16 ± 2.96	7.98 ± 2.77	8.61 ± 2.32
Sweden	7.43 ± 2.89	9.01 ± 3.11	6.64 ± 2.25	8.04 ± 2.67	7.86 ± 3.25	8.58 ± 3.32	9.12 ± 3.17	8.45 ± 3.39
Finland	8.05 ± 3.47	7.84 ± 2.48	7.84 ± 3.20	7.78 ± 3.31	6.35 ± 1.99	6.58 ± 2.74	6.30 ± 1.72	6.01 ± 2.14
Greece	8.29 ± 3.10	7.10 ± 2.30	9.98 ± 3.77	7.81 ± 2.91	8.60 ± 6.27	7.78 ± 2.60	10.31 ± 2.36	8.35 ± 2.92
South Africa	8.63 ± 1.61	10.38 ± 1.53	9.37 ± 3.48	7.06 ± 3.19	6.71 ± 3.78	10.31 ± 4.19	8.00 ± 4.50	6.10 ± 2.32
Brazil	7.31 ± 2.50	8.41 ± 3.06	7.60 ± 2.82	10.22 ± 3.11	7.94 ± 3.19	7.34 ± 3.03	9.36 ± 2.94	7.78 ± 2.68
Mexico	9.04 ± 3.93	10.32 ± 2.81	9.71 ± 6.11	9.19 ± 3.75	9.57 ± 3.57	8.97 ± 3.36	8.01 ± 3.50	6.99 ± 3.16
Argentina	10.55	5.85	9.55 ± 3.14	7.25 ± 3.00	4.63 ± 0.21	11.09 ± 3.30	11.16 ± 0.53	8.67 ± 3.14
India	5.40	10.78 ± 3.92	6.87 ± 2.68	6.38 ± 2.90	9.44 ± 1.51	9.65 ± 0.17	8.22 ± 3.74	8.58 ± 2.95
Israel	8.7 ± 2.60	8.93 ± 4.48	8.78 ± 3.51	10.09 ± 3.80	7.13 ± 2.68	8.91 ± 2.18	9.42 ± 3.68	10.21 ± 4.32
Slovenia			9.50 ± 3.82	4.58	10.82	7.16 ± 3.24	8.06 ± 4.48	

Data for Non-African runners are sorted in order of the number of finishers of each country

**Table 3:** Results of the mixed-effects regression analyses for change in running speed in female half-marathoners across years

Parameter	Estimate	SE	DF	T	*p* value	95 % CI	
						Upper	Lower
Ethiopia							
Constant term	293.167388	371.574985	43.863	0.789	0.434	−455.758951	1042.093726
Year	−0.140332	0.184932	43.873	−0.759	0.452	−0.513068	0.232405
Cage	−0.173148	0.135294	36.958	−1.280	0.209	−0.447291	0.100995
Cage^2^	−0.004387	0.006022	27.717	−0.728	0.472	−0.016727	0.007954
Kenya							
Constant term	−21.426837	150.844832	36.330	−.0142	0.888	−327.257984	284.404310
Year	0.017207	0.075087	36.326	0.229	0.820	−0.135029	0.169443
Cage	−0.144332	0.060829	27.148	−2.373	0.025	−0.269111	−0.019552
Cage^2^	−0.003215	0.003653	30.133	−0.880	0.386	−0.010674	0.004243
Austria							
Constant term	31.578045	29.443671	597.888	1.072	0.284	−26.247547	89.403637
Year	−0.011598	0.014658	597.871	−0.791	0.429	−0.040386	0.017190
Cage	−0.028602	0.006916	600.177	−4.136	0.000	−0.042184	−0.015021
Cage^2^	0.000663	0.000536	608.882	1.237	0.217	−0.000390	0.001716
Canada							
Constant term	−125.514888	73.115253	155.945	−1.717	0.088	−269.938931	18.909154
Year	0.066249	0.036396	155.933	1.820	0.071	−0.005644	0.138143
Cage	−0.020679	0.014519	170.268	−1.424	0.156	−0.049340	0.007982
Cage^2^	7.822872E−5	0.001161	162.137	0.067	0.946	−0.002214	0.002370
Czech Republic							
Constant term	−220.270108	155.839548	40.611	−1.413	0.165	−535.086061	94.545845
Year	0.113694	0.077609	40.610	1.465	0.151	−0.043086	0.270474
Cage	−0.080535	0.051903	45.473	−1.552	0.128	−0.185044	0.023974
Cage^2^	0.003476	0.004529	46.743	0.768	0.447	−0.005637	0.012589
Denmark							
Constant term	−144.099673	142.620810	55.173	−1.010	0.317	−429.897999	141.698652
Year	0.076052	0.070989	55.174	1.071	0.289	−0.066203	0.218308
Cage	−.012618	0.030636	58.407	−0.412	0.682	−0.073934	0.048697
Cage^2^	−.000446	0.002495	59.214	−0.179	0.859	−0.005439	0.004546
Spain							
Constant term	−5.366288	67.028907	141.702	−0.080	0.936	−137.872163	127.139587
Year	0.007615	0.033366	141.702	0.228	0.820	−0.058344	0.073574
Cage	0.001968	0.014710	129.823	0.134	0.894	−0.027134	0.031069
Cage^2^	−0.000865	0.001169	144.790	−0.740	0.461	−0.003175	0.001445
France							
Constant term	51.171466	15.295732	4653.214	3.345	0.001	21.184581	81.158350
Year	−0.020670	0.007613	4653.140	−2.715	0.007	−0.035595	−0.005744
Cage	−0.023120	0.003408	4755.183	−6.783	0.000	−0.029801	−0.016438
Cage^2^	−0.000602	0.000252	4810.855	−2.392	0.017	−0.001095	−0.000109
Great Britain							
Constant term	28.901729	42.017402	749.113	0.688	0.492	−53.584137	111.387595
Year	−0.009779	0.020917	749.099	−0.468	0.640	−0.050842	0.031283
Cage	0.009513	0.009652	844.541	0.986	0.325	−0.009432	0.028457
Cage^2^	−0.001723	0.000775	871.578	−2.222	0.027	−0.003245	−0.000201
Germany							
Constant term	15.158368	14.956731	4456.047	1.013	0.311	−14.164251	44.480986
Year	−0.003244	0.007446	4455.945	−0.436	0.663	−0.017841	0.011353
Cage	−0.023479	0.002955	3893.061	−7.946	0.000	−0.029272	−0.017686
Cage^2^	−0.000163	0.000198	3910.190	−0.822	0.411	−0.000551	0.000225
Italy							
Constant term	55.277603	39.067728	844.859	1.415	0.157	−21.403589	131.958795
Year	−0.022114	0.019449	844.831	−1.137	0.256	−0.060287	0.016060
Cage	−0.049367	0.010292	954.174	−4.797	0.000	−0.069565	−0.029169
Cage^2^	−0.001914	0.000776	961.174	−2.467	0.014	−0.003436	−0.000391
Japan							
Constant term	70.707877	63.986213	101.377	1.105	0.272	−56.217842	197.633596
Year	−0.031946	0.031855	101.405	−1.003	0.318	−.0095133	0.031242
Cage	−0.047542	0.017703	159.965	−2.685	0.008	−0.082504	−0.012579
Cage^2^	0.001527	0.000930	166.855	1.641	0.103	−0.000310	0.003363
Principality of Liechtenstein							
Constant term	10.483455	64.869741	255.178	0.162	0.872	−117.264786	138.231696
Year	0.000233	0.032291	255.155	0.007	0.994	−0.063358	0.063823
Cage	−0.012747	0.013132	275.553	−0.971	0.333	−0.038598	0.013105
Cage^2^	−0.001544	0.001094	290.811	−1.411	0.159	−0.003697	0.000610
Luxembourg							
Constant term	−47.666634	97.322485	48.909	−0.490	0.626	−243.252737	147.919469
Year	0.027753	0.048451	48.906	0.573	0.569	−0.069617	0.125123
Cage	−0.023725	0.013396	31.814	−1.771	0.086	−0.051017	0.003567
Cage^2^	0.001603	0.001046	30.499	1.533	0.136	−0.000532	0.003737
Netherlands							
Constant term	133.938661	125.455169	149.777	1.068	0.287	−113.951882	381.829205
Year	−0.062003	0.062457	149.769	−0.993	0.322	−0.185413	0.061407
Cage	−0.011427	0.021283	105.363	−0.537	0.592	−0.053626	0.030771
Cage^2^	0.000320	0.001177	84.597	0.272	0.786	−0.002020	0.002660
Norway							
Constant term	−487.272616	173.193914	54.820	−2.813	0.007	−834.386566	−140.158666
Year	0.247478	0.086290	54.821	2.868	0.006	0.074537	0.420419
Cage	−0.115170	0.020554	36.162	−5.603	0.000	−0.156850	−0.073491
Cage^2^	0.002664	0.001240	33.459	2.150	0.039	0.000144	0.005185
Portugal							
Constant term	−474.101901	231.004145	55.403	−2.052	0.045	−936.968950	−11.234851
Year	0.241610	0.114974	55.397	2.101	0.040	0.011234	0.471987
Cage	−0.006509	0.059010	54.339	−0.110	0.913	−0.124800	0.111783
Cage^2^	0.004436	0.005816	53.398	0.763	0.449	−0.007227	0.016100
Switzerland							
Constant term	17.415465	3.082751	85,158.813	5.649	0.000	11.373300	23.457631
Year	−0.003429	0.001535	85,155.422	−2.235	0.025	−0.006437	−0.000422
Cage	−0.010236	0.000499	71,948.591	−20.518	0.000	−0.011214	−0.009259
Cage^2^	−0.000417	3.413093E−5	70,505.318	−12.217	0.000	−0.000484	−0.000350
United States of America							
Constant term	−102.180938	71.308524	295.142	−1.433	0.153	−242.518553	38.156677
Year	0.055053	0.035488	295.135	1.551	0.122	−0.014789	0.124895
Cage	−0.011404	0.012867	301.169	−0.886	0.376	−0.036725	0.013917
Cage^2^	0.001037	0.000928	305.711	1.118	0.265	−0.000789	0.002863
Australia							
Constant term	535.990276	98.301322	13.686	5.453	0.000	324.700562	747.279990
Year	−0.262740	0.048925	13.688	−5.370	0.000	−0.367899	−0.157580
Cage	0.063641	0.055873	47.462	1.139	0.260	−0.048733	0.176014
Cage^2^	−0.002360	0.002842	46.702	−0.830	0.411	−0.008078	0.003358
Belgium							
Constant term	7.038611	107.628613	165.205	0.065	0.948	−205.466290	219.543511
Year	0.000697	0.053552	165.225	0.013	0.990	−0.105038	0.106433
Cage	0.000626	0.015637	117.693	0.040	0.968	−0.030341	0.031593
Cage^2^	0.000700	0.001032	108.364	0.678	0.499	−0.001345	0.002745
Hungary							
Constant term	−275.257629	154.694094	51.000	−1.779	0.081	−585.818982	35.303724
Year	0.142544	0.076947	51.000	1.853	0.070	−0.011933	0.297021
Cage	−0.083478	0.047940	51.000	−1.741	0.088	−0.179721	0.012765
Cage^2^	0.000717	0.002086	51.000	0.344	0.732	−0.003470	0.004904
Ireland							
Constant term	−33.859940	165.743508	57.989	−0.204	0.839	−365.633001	297.913121
Year	0.021718	0.082524	57.993	0.263	0.793	−0.143473	0.186909
Cage	−0.036636	0.037101	37.244	−0.987	0.330	−0.111793	0.038521
Cage^2^	−0.000562	0.003459	32.491	−0.162	0.872	−0.007603	0.006480
Poland							
Constant term	−72.726997	104.051935	60.961	−0.699	0.487	−280.794379	135.340384
Year	0.040347	0.051798	60.974	0.779	0.439	−0.063230	0.143924
Cage	−0.053476	0.023476	65.801	−2.278	0.026	−0.100351	−0.006601
Cage^2^	−0.001860	0.001865	69.955	−0.997	0.322	−0.005579	0.001859
Russia							
Constant term	−106.441127	155.565229	42.567	−0.684	0.498	−420.260572	207.378318
Year	0.057206	0.077443	42.538	0.739	0.464	−0.099022	0.213434
Cage	−0.072584	0.042398	55.257	−1.712	0.093	−0.157542	0.012374
Cage^2^	0.004846	0.003169	47.538	1.529	0.133	−0.001528	0.011219
Sweden							
Constant term	75.820147	143.966277	104.839	0.527	0.600	−209.643494	361.283787
Year	−0.033856	0.071641	104.838	−0.473	0.637	−0.175909	0.108197
Cage	−0.016537	0.020415	67.482	−0.810	0.421	−0.057279	0.024206
Cage^2^	0.001067	0.001713	108.162	0.623	0.535	−0.002328	0.004462
Finland							
Constant term	−46.926724	100.378904	55.066	−0.467	0.642	−248.085146	154.231697
Year	0.027006	0.049943	55.068	0.541	0.591	−0.073079	0.127091
Cage	−0.053711	0.015750	46.121	−3.410	0.001	−0.085411	−0.022011
Cage^2^	0.001208	0.001076	44.445	1.123	0.268	−0.000960	0.003376
Greece							
Constant term	−105.607974	339.956176	18.425	−0.311	0.760	−818.651669	607.435721
Year	0.056703	0.169175	18.428	0.335	0.741	−0.298130	0.411535
Cage	0.011301	0.080214	18.335	0.141	0.889	−0.157002	0.179604
Cage^2^	0.005226	0.004315	20.000	1.211	0.240	−0.003776	0.014227
Republic of South Africa							
Constant term	195.281269	261.100120	30.776	0.748	0.460	−337.393318	727.955856
Year	−0.093079	0.130031	30.771	−0.716	0.479	−0.358359	0.172201
Cage	−0.033597	0.065427	31.996	−0.514	0.611	−0.166869	0.099674
Cage^2^	0.000214	0.003268	27.237	0.066	0.948	−0.006488	0.006916
Brazil							
Constant term	178.944876	212.772674	32.077	0.841	0.407	−254.418110	612.307863
Year	−0.084760	0.105910	32.083	−0.800	0.429	−0.300469	0.130949
Cage	−0.054561	0.036085	32.952	−1.512	0.140	−0.127981	0.018860
Cage^2^	0.003708	0.002066	37.833	1.795	0.081	−0.000475	0.007891
Mexico							
Constant term	−208.359308	343.548300	13.049	−0.606	0.555	−950.268215	533.549600
Year	0.108288	0.170848	13.048	0.634	0.537	−0.260670	0.477246
Cage	0.017053	0.033477	1.149	0.509	0.691	−0.297964	0.332070
Cage^2^	−0.004284	0.002132	1.006	−2.009	0.293	−0.031003	0.022435
Argentina							
Constant term	171.161142	449.529264	12.527	0.381	0.710	−803.726404	1146.048688
Year	−0.080710	0.223646	12.527	−0.361	0.724	−0.565727	0.404306
Cage	−0.121342	0.107487	7.809	−1.129	0.292	−0.370266	0.127582
Cage^2^	0.004002	0.011220	5.403	0.357	0.735	−0.024206	0.032210
India							
Constant term	−180.991329	118.481416	23.000	−1.528	0.140	−426.088812	64.106153
Year	0.095159	0.058967	23.000	1.614	0.120	−0.026822	0.217141
Cage	0.034212	0.026224	23.000	1.305	0.205	−0.020037	0.088460
Cage^2^	0.002970	0.002305	23.000	1.288	0.210	−0.001798	0.007737
Israel							
Constant term	1791.341424	749.677965	4.526	2.389	0.068	−198.063460	3780.746308
Year	−0.884457	0.372205	4.522	−2.376	0.069	−1.872464	0.103550
Cage	−0.130351	0.107475	8.468	−1.213	0.258	−0.375823	0.115121
Cage^2^	−0.026869	0.011891	11.589	−2.260	0.044	−0.052880	−0.000858
Slovenia							
Constant term	−31.714993	134.226844	13.547	−0.236	0.817	−320.508524	257.078539
Year	0.020363	0.066884	13.547	0.304	0.765	−0.123540	0.164266
Cage	−0.084123	0.046744	8.409	−1.800	0.108	−0.191009	0.022763
Cage^2^	−0.001050	0.002818	9.076	−0.373	0.718	−0.007416	0.005317

Data for Non-African runners are sorted in order of the number of finishers of each country

*Cage* centered age, *Cage* ^2^ centered age squared

**Table 4:** Results of the mixed-effects regression analyses for change in running speed in male half-marathoners across years

Parameter	Estimate	SE	DF	T	*p* value	95 % CI	
						Upper	Lower
Ethiopia							
Constant term	98.099117	174.871376	30.494	0.561	0.579	−258.793601	454.991835
Year	−0.043552	0.087058	30.500	−0.500	0.620	−0.221226	0.134123
Cage	−0.250494	0.185335	45.246	−1.352	0.183	−0.623723	0.122734
Cage^2^	−0.012719	0.007260	42.721	−1.752	0.087	−0.027363	0.001925
Kenya							
Constant term	88.307393	103.114750	61.076	0.856	0.395	−117.878135	294.492921
Year	−0.037470	0.051349	61.091	−0.730	0.468	−0.140145	0.065205
Cage	−0.066120	0.029956	133.511	−2.207	0.029	−0.125371	−0.006870
Cage^2^	−0.002035	0.001386	133.757	−1.469	0.144	−0.004776	0.000706
Austria							
Constant term	8.193904	15.718906	1374.455	0.521	0.602	−22.641739	39.029547
Year	0.000216	0.007826	1374.306	0.028	0.978	−0.015136	0.015568
Cage	−0.016008	0.004315	1443.906	−3.710	0.000	−0.024471	−0.007544
Cage^2^	−0.000501	0.000294	1458.820	−1.702	0.089	−0.001078	7.633939E−5
Canada							
Constant term	−21.298964	52.088221	337.247	−0.409	0.683	−123.757696	81.159769
Year	0.014351	0.025943	337.200	0.553	0.580	−0.036679	0.065382
Cage	−0.024635	0.011096	359.294	−2.220	0.027	−0.046457	−0.002814
Cage^2^	0.000544	0.000695	362.647	0.783	0.434	−0.000822	0.001910
Czech Republic							
Constant term	154.008680	81.928758	100.744	1.880	0.063	−8.520953	316.538313
Year	−0.072109	0.040820	100.777	−1.767	0.080	−0.153086	0.008869
Cage	−0.003265	0.013101	75.567	−0.249	0.804	−0.029361	0.022830
Cage^2^	−0.002162	0.000996	78.010	−2.171	0.033	−0.004144	−0.000179
Denmark							
Constant term	73.075047	85.575129	152.801	0.854	0.394	−95.988101	242.138195
Year	−0.032295	0.042595	152.782	−0.758	0.450	−0.116447	0.051857
Cage	0.004069	0.013983	120.832	0.291	0.772	−0.023615	0.031752
Cage^2^	−0.002067	0.000851	124.782	−2.429	0.017	−0.003752	−0.000383
Spain							
Constant term	73.070026	58.997907	419.804	1.239	0.216	−42.898085	189.038138
Year	−0.031824	0.029368	419.811	−1.084	0.279	−0.089550	0.025902
Cage	−0.015581	0.013136	435.510	−1.186	0.236	−0.041399	0.010238
Cage^2^	0.001634	0.000911	471.708	1.793	0.074	−0.000156	0.003425
France							
Constant term	2.152551	8.424878	11,440.730	0.255	0.798	−14.361653	18.666754
Year	0.003734	0.004195	11,440.223	0.890	0.373	−0.004488	0.011956
Cage	−0.024520	0.002065	11,842.861	−11.874	0.000	−0.028568	−0.020472
Cage^2^	−0.000555	0.000144	11,634.921	−3.864	0.000	−0.000837	−0.000274
Great Britain							
Constant term	37.386616	22.952563	1729.593	1.629	0.104	−7.631084	82.404316
Year	−0.014237	0.011430	1729.576	−1.246	0.213	−0.036654	0.008181
Cage	−0.007171	0.005013	1775.835	−1.430	0.153	−0.017003	0.002662
Cage^2^	−0.000507	0.000363	1811.205	−1.399	0.162	−0.001218	0.000204
Germany							
Constant term	26.340398	7.339500	12,201.868	3.589	0.000	11.953816	40.726980
Year	−0.008802	0.003654	12,201.252	−2.409	0.016	−0.015965	−0.001639
Cage	−0.015672	0.001614	11,817.685	−9.708	0.000	−0.018836	−0.012508
Cage^2^	−0.000337	0.000106	11,885.807	−3.164	0.002	−0.000545	−0.000128
Italy							
Constant term	32.563297	20.328196	2372.545	1.602	0.109	−7.299571	72.426164
Year	−0.010999	0.010122	2372.366	−1.087	0.277	−0.030847	0.008850
Cage	−0.045951	0.005681	2514.295	−8.089	0.000	−0.057090	−0.034812
Cage^2^	−0.000765	0.000380	2470.087	−2.015	0.044	−0.001510	−2.039314E−5
Japan							
Constant term	14.867692	48.121533	316.697	0.309	0.758	−79.810599	109.545984
Year	−.003770	0.023954	316.650	−.157	0.875	−.050900	0.043360
Cage	−.058721	0.010081	397.745	−5.825	0.000	−.078540	−.038902
Cage^2^	4.080624E−5	0.000449	341.270	0.091	0.928	−.000843	0.000924
Fürstentum Liechtenstein							
Constant term	36.488409	42.462409	604.502	0.859	0.391	−46.903349	119.880168
Year	−0.012766	0.021142	604.548	−0.604	0.546	−0.054287	0.028756
Cage	−0.001820	0.009507	565.166	−0.191	0.848	−0.020492	0.016853
Cage^2^	−0.001896	0.000777	592.741	−2.438	0.015	−0.003422	−0.000369
Luxembourg							
Constant term	−15.253456	80.615224	92.293	−0.189	0.850	−175.355473	144.848561
Year	0.011431	0.040137	92.287	0.285	0.776	−0.068280	0.091143
Cage	−0.065466	0.021046	112.887	−3.111	0.002	−0.107162	−0.023770
Cage^2^	0.002067	0.001460	76.329	1.415	0.161	−0.000842	0.004975
Netherlands							
Constant term	43.412228	54.590318	317.931	0.795	0.427	−63.991689	150.816144
Year	−0.016855	0.027175	317.960	−0.620	0.536	−0.070321	0.036612
Cage	−0.029740	0.011819	303.713	−2.516	0.012	−0.052998	−0.006482
Cage^2^	−0.001104	0.000799	294.009	−1.382	0.168	−0.002675	0.000468
Norway							
Constant term	−108.473099	87.956547	66.260	−1.233	0.222	−284.071127	67.124929
Year	0.058583	0.043794	66.285	1.338	0.186	−0.028848	0.146014
Cage	0.003335	0.021397	98.282	0.156	0.876	−0.039125	0.045796
Cage^2^	−0.001692	0.001159	78.629	−1.460	0.148	−0.004000	0.000615
Portugal							
Constant term	67.632099	92.672293	171.225	0.730	0.467	−115.295174	250.559373
Year	−0.028137	0.046162	171.263	−0.610	0.543	−0.119256	0.062982
Cage	0.009515	0.023055	186.876	0.413	0.680	−0.035968	0.054997
Cage^2^	−0.001022	0.001966	185.843	−0.520	0.604	−0.004900	0.002855
Switzerland							
Constant term	8.817285	1.667403	223,427.050	5.288	0.000	5.549219	12.085351
Year	0.000791	0.000830	223,414.735	0.953	0.340	−0.000836	0.002418
Cage	−0.010964	0.000294	204,230.290	−37.270	0.000	−0.011540	−0.010387
Cage^2^	−0.000368	2.032780E−5	202,092.789	−18.089	0.000	−0.000408	−0.000328
United States of America							
Constant term	54.392263	32.424867	708.442	1.677	0.094	−9.268067	118.052594
Year	−0.023017	0.016143	708.387	−1.426	0.154	−0.054711	0.008677
Cage	−0.009382	0.006245	705.174	−1.502	0.133	−0.021643	0.002878
Cage^2^	−0.000283	0.000441	689.741	−0.641	0.521	−0.001149	0.000583
Australia							
Constant term	52.302602	107.606779	108.032	0.486	0.628	−160.991990	265.597194
Year	−0.021678	0.053582	108.089	−0.405	0.687	−0.127885	0.084529
Cage	−0.013969	0.030696	133.451	−0.455	0.650	−0.074683	0.046745
Cage^2^	−0.000317	0.002070	136.856	−0.153	0.878	−0.004411	0.003776
Belgium							
Constant term	107.524420	49.224327	501.956	2.184	0.029	10.813323	204.235517
Year	−0.049210	0.024501	501.911	−2.008	0.045	−0.097348	−0.001073
Cage	−0.033350	0.009176	447.125	−3.635	0.000	−0.051383	−0.015317
Cage^2^	−7.340408E−5	0.000666	417.563	−0.110	0.912	−0.001382	0.001235
Hungary							
Constant term	5.737883	94.942100	159.346	0.060	0.952	−181.769287	193.245052
Year	0.002193	0.047257	159.320	0.046	0.963	−0.091137	0.095523
Cage	−0.021118	0.020068	158.936	−1.052	0.294	−0.060752	0.018515
Cage^2^	0.001436	0.001087	174.946	1.320	0.188	−0.000710	0.003582
Ireland							
Constant term	138.624168	77.025730	130.753	1.800	0.074	−13.753783	291.002120
Year	−0.064935	0.038349	130.724	−1.693	0.093	−0.140799	0.010930
Cage	0.012905	0.020461	147.172	0.631	0.529	−0.027530	0.053340
Cage^2^	0.001772	0.001597	146.087	1.110	0.269	−0.001384	0.004928
Poland							
Constant term	86.086931	104.182157	192.160	0.826	0.410	−119.400506	291.574369
Year	−0.038832	0.051868	192.148	−0.749	0.455	−0.141137	0.063472
Cage	−0.092905	0.018121	167.220	−5.127	0.000	−0.128680	−0.057130
Cage^2^	0.001517	0.001224	171.169	1.239	0.217	−0.000899	0.003933
Russia							
Constant term	211.097501	112.913539	96.255	1.870	0.065	−13.026515	435.221517
Year	−0.100778	0.056233	96.273	−1.792	0.076	−0.212397	0.010840
Cage	−0.066677	0.024282	81.019	−2.746	0.007	−0.114991	−0.018364
Cage^2^	−0.004585	0.001957	62.648	−2.343	0.022	−0.008497	−0.000674
Sweden							
Constant term	23.098100	70.780786	194.032	0.326	0.745	−116.500399	162.696600
Year	−0.007622	0.035234	193.992	−0.216	0.829	−0.077113	0.061870
Cage	−0.041879	0.012513	177.901	−3.347	0.001	−0.066573	−0.017186
Cage^2^	−0.000214	0.000893	193.338	−0.239	0.811	−0.001976	0.001548
Finland							
Constant term	8.266275	52.736089	163.256	0.157	0.876	−95.866485	112.399036
Year	−0.000566	0.026249	163.232	−0.022	0.983	−0.052397	0.051266
Cage	−0.041553	0.011615	170.064	−3.578	0.000	−0.064481	−0.018625
Cage^2^	0.000222	0.000718	147.558	0.309	0.758	−0.001197	0.001640
Greece							
Constant term	−61.629272	138.778354	63.917	−0.444	0.658	−338.877861	215.619317
Year	0.034774	0.069126	63.920	0.503	0.617	−0.103324	0.172871
Cage	−0.045672	0.029991	67.495	−1.523	0.132	−0.105525	0.014181
Cage^2^	0.001362	0.002210	66.813	0.616	0.540	−0.003050	0.005774
Republic of South Africa							
Constant term	97.835143	206.579371	37.822	0.474	0.639	−320.427710	516.097997
Year	−0.045155	0.102815	37.832	−0.439	0.663	−0.253323	0.163012
Cage	−0.114764	0.045331	42.716	−2.532	0.015	−0.206201	−0.023327
Cage^2^	0.002014	0.003936	43.893	0.512	0.611	−0.005919	0.009947
Brazil							
Constant term	69.573202	122.101031	76.685	0.570	0.570	−173.576935	312.723340
Year	−0.030992	0.060774	76.687	−0.510	0.612	−0.152015	0.090032
Cage	−0.005665	0.035651	84.804	−0.159	0.874	−0.076551	0.065221
Cage^2^	0.000800	0.002584	85.927	0.310	0.758	−0.004337	0.005937
Mexico							
Constant term	41.878671	159.784287	57.808	0.262	0.794	−277.986904	361.744245
Year	−0.016374	0.079565	57.848	−0.206	0.838	−0.175650	0.142902
Cage	0.004652	0.037631	61.634	0.124	0.902	−0.070580	0.079884
Cage^2^	0.000324	0.002948	56.403	0.110	0.913	−0.005580	0.006229
Argentina							
Constant term	−280.869673	275.764919	14.061	−1.019	0.326	−872.087719	310.348372
Year	0.143819	0.137206	14.060	1.048	0.312	−0.150341	0.437979
Cage	−0.105665	0.095288	20.398	−1.109	0.280	−0.304184	0.092855
Cage^2^	−0.008941	0.013370	20.476	−0.669	0.511	−0.036788	0.018907
India							
Constant term	184.538334	187.481998	40.972	0.984	0.331	−194.097033	563.173702
Year	−0.087437	0.093418	40.964	−0.936	0.355	−0.276104	0.101229
Cage	−0.043125	0.036785	44.595	−1.172	0.247	−0.117232	0.030982
Cage^2^	−0.000763	0.003355	41.543	−0.228	0.821	−0.007536	0.006009
Israel							
Constant term	23.126668	168.388173	45.039	0.137	0.891	−316.016346	362.269682
Year	−0.006935	0.083826	45.030	−0.083	0.934	−0.175766	0.161896
Cage	−0.016089	0.022043	28.717	−0.730	0.471	−0.061192	0.029014
Cage^2^	−0.000399	0.001451	29.170	−0.275	0.785	−0.003365	0.002567
Slovenia							
Constant term	449.153792	328.346727	19.574	1.368	0.187	−236.722979	1135.030562
Year	−0.219405	0.163419	19.579	−1.343	0.195	−0.560762	0.121952
Cage	−0.078618	0.063055	11.412	−1.247	0.237	−0.216792	0.059557
Cage^2^	0.003187	0.005660	11.316	0.563	0.584	−0.009229	0.015603

Data for Non-African runners are sorted in order of the number of finishers of each country

*Cage* centered age, *Cage* ^2^ centered age squared

**Table 5:** Running speed (km/h) with mean ± SD for female and male East-African and Non-African marathoners

	1999	2000	2001	2002	2003	2004	2005	2006
Women								
Ethiopia							18.64	
Kenya						18.26		
Austria	16.42 ± 1.62	18.32 ± 1.81	12.34	13.83 ± 5.78	12.36 ± 2.18	9.80 ± 0.21	12.65 ± 3.26	12.39 ± 2.43
France	13.70 ± 4.13	13.77 ± 4.01	13.83 ± 4.05	13.72 ± 3.88	12.42 ± 3.98	13.38 ± 3.97	13.82 ± 4.43	13.27 ± 4.05
Great Britain		10.50 ± 1.45	12.73 ± 4.45	13.14 ± 5.02	12.96 ± 3.95	13.46 ± 4.35	14.47 ± 4.58	12.72 ± 2.65
Germany	11.68 ± 2.24	11.17 ± 2.63	13.90 ± 3.79	12.20 ± 3.77	12.63 ± 3.78	12.11 ± 3.34	13.04 ± 3.68	13.00 ± 3.60
Italy	17.10	19.19 ± 0.52	19.64 ± 1.30	12.67 ± 5.70	11.93 ± 3.77	15.39 ± 4.39	12.06 ± 4.28	18.78 ± 1.77
Japan	14.13	10.96	11.72 ± 5.02	7.88 ± 1.11	18.28	13.34 ± 4.69	14.27 ± 5.01	18.70
Switzerland	14.46 ± 4.34	15.30 ± 4.19	14.74 ± 4.13	15.03 ± 4.06	15.60 ± 4.01	15.44 ± 4.09	15.08 ± 4.14	15.20 ± 4.12
Canada				9.31	10.29 ± 5.82	8.00 ± 1.63	12.51	12.85 ± 7.29
Liechtenstein		11.65	9.92 ± 2.39		16.05 ± 5.43		18.13 ± 1.01	19.75 ± 0.15
USA		17.95				17.17 ± 4.07	13.92 ± 4.10	9.85 ± 1.43
Belgium	10.77				19.87 ± 0.49	10.95 ± 0.37	11.65 ± 1.62	10.90 ± 0.79
Spain							12.48	
Poland				11.24 ± 0.64	8.85			
Men								
Ethiopia			17.47 ± 2.28					
Kenya		18.81	17.95 ± 1.44		17.61 ± 1.97	17.43 ± 1.62	17.28	
Austria			12.66 ± 0.09	9.10	19.37	16.18	14.40 ± 7.02	15.59 ± 5.27
France	14.08 ± 4.06	13.47 ± 3.67	13.17 ± 3.75	13.17 ± 3.30	13.28 ± 3.79	13.41 ± 3.84	13.50 ± 3.89	12.87 ± 3.48
Great Britain	9.27 ± 0.96	12.54 ± 3.61	12.01 ± 3.57	15.21 ± 2.94	15.02 ± 4.05	14.08 ± 3.79	14.15 ± 4.51	13.26 ± 3.82
Germany	12.51 ± 3.32	13.02 ± 3.78	12.67 ± 3.28	12.60 ± 3.48	12.89 ± 3.70	13.00 ± 3.66	12.49 ± 3.55	12.89 ± 3.65
Italy	16.23 ± 4.37	12.51 ± 3.77	12.85 ± 2.99	12.49 ± 4.01	12.50 ± 3.24	14.23 ± 4.04	13.66 ± 3.90	15.14 ± 3.86
Japan	15.09	14.42	12.84 ± 5.70	11.47	11.45 ± 3.85	11.09 ± 4.17	12.92 ± 4.63	11.53 ± 3.41
Switzerland	14.41 ± 3.99	14.84 ± 4.09	14.73 ± 4.07	14.77 ± 4.09	14.93 ± 4.11	14.71 ± 4.08	14.76 ± 4.04	14.83 ± 4.06
Canada	14.00 ± 0.72	18.98	12.22 ± 4.96	12.37 ± 4.83	10.50 ± 3.91	10.90 ± 3.36	11.12 ± 4.09	13.14 ± 4.06
Liechtenstein			18.70	17.56 ± 1.45	19.11 ± 1.21	15.41 ± 4.16	17.42 ± 3.08	17.80 ± 1.43
USA	12.39 ± 3.85	10.79 ± 1.55	11.25 ± 3.38	13.62 ± 3.72	12.12 ± 3.55	12.65 ± 4.21	12.14 ± 3.48	12.86 ± 4.29
Belgium		12.23		14.97 ± 5.32	14.80 ± 4.67	13.65 ± 4.98	12.71 ± 4.02	12.70 ± 3.91
Spain		13.08	19.33	18.87 ± 1.06	13.90 ± 5.05	12.63 ± 2.21	13.37 ± 4.71	14.10 ± 2.28
Poland		10.07		11.65 ± 1.73	9.05	10.36 ± 2.31	9.73	17.70 ± 2.29

	2007	2008	2009	2010	2011	2012	2013	2014
Women								
Ethiopia					18.78	19.24		
Kenya					18.49			
Austria	11.84 ± 2.64	12.88 ± 3.92	12.48 ± 1.87	14.49 ± 4.76	11.09 ± 1.30	13.21 ± 3.31	11.29 ± 2.23	11.40 ± 2.61
France	13.09 ± 3.75	14.14 ± 4.36	13.60 ± 4.05	13.04 ± 3.72	13.37 ± 3.81	13.48 ± 3.89	14.01 ± 3.65	13.47 ± 4.22
Great Britain	14.06 ± 4.43	12.85 ± 4.03	13.15 ± 0.79	13.31 ± 4.35	14.81 ± 4.85	11.46 ± 5.80	14.10 ± 3.80	12.67 ± 3.57
Germany	13.71 ± 3.85	12.97 ± 3.48	12.89 ± 3.93	13.47 ± 4.39	12.81 ± 3.56	13.24 ± 3.62	13.45 ± 4.25	12.94 ± 3.87
Italy	16.34 ± 3.92	17.44 ± 3.70	12.78 ± 3.09	18.82 ± 1.04	17.47 ± 1.84	16.90 ± 4.52	18.56 ± 0.80	16.05 ± 4.56
Japan	13.33 ± 4.01	14.24 ± 4.74	19.90	13.66 ± 1.15	11.80 ± 4.36	18.00 ± 0.39	18.49 ± 0.20	19.47 ± 1.09
Switzerland	15.20 ± 4.07	14.90 ± 4.16	15.21 ± 4.08	14.98 ± 4.27	15.03 ± 4.09	14.72 ± 4.14	15.24 ± 4.14	14.76 ± 4.02
Canada	17.99	18.53 ± 0.86	14.73 ± 6.33	9.44 ± 1.67	12.47 ± 3.63	8.06 ± 2.00	12.10 ± 3.62	11.97 ± 6.90
Liechtenstein	18.78 ± 0.79	18.31 ± 2.04	16.96 ± 4.11	12.09	16.55	16.95 ± 3.38		18.56 ± 0.24
USA	12.12 ± 4.17	13.84 ± 3.76	10.03 ± 0.19	9.39 ± 0.59	10.36 ± 1.59	15.87		12.08 ± 5.31
Belgium	11.66	9.07		13.07	9.30			
Spain	17.96 ± 1.64	10.75	12.95 ± 1.50		11.79 ± 1.48	12.19 ± 0.21	19.09	12.79 ± 1.39
Poland			12.25	10.67	11.69 ± 0.57	18.01 ± 0.67	15.50 ± 3.96	12.10 ± 1.93
Men								
Ethiopia	15.67 ± 3.28	16.26	17.34 ± 1.61	15.87 ± 0.21	15.13		15.91 ± 1.04	15.18
Kenya	16.88 ± 2.65	18.43 ± 0.25	18.50 ± 0.19	18.10 ± 0.58	17.49 ± 0.47	17.96	18.15 ± 1.28	17.47 ± 1.68
Austria	17.87 ± 0.91	15.28 ± 5.19		10.60			20.29	12.44 ± 3.74
France	13.12 ± 3.79	13.69 ± 4.00	13.61 ± 3.96	13.21 ± 3.77	13.21 ± 3.67	13.21 ± 3.86	13.34 ± 3.66	13.70 ± 3.82
Great Britain	13.08 ± 4.04	13.28 ± 4.01	12.66 ± 3.74	13.39 ± 3.95	12.97 ± 3.97	12.64 ± 3.82	13.63 ± 3.90	12.48 ± 3.49
Germany	13.05 ± 3.77	12.64 ± 3.50	12.93 ± 3.62	13.07 ± 3.80	12.98 ± 3.69	13.07 ± 3.70	13.07 ± 3.54	13.27 ± 3.93
Italy	14.54 ± 4.07	15.13 ± 4.17	14.34 ± 4.05	12.79 ± 3.71	12.93 ± 4.05	13.30 ± 4.03	13.40 ± 4.10	12.38 ± 3.35
Japan	11.22 ± 2.74	12.79 ± 3.82	11.81 ± 4.02	10.66 ± 3.95	11.61 ± 4.59	11.73 ± 4.31	14.45 ± 5.00	12.46 ± 7.03
Switzerland	14.78 ± 4.07	14.87 ± 4.09	14.77 ± 4.08	14.83 ± 4.11	14.87 ± 4.12	14.75 ± 4.01	14.89 ± 4.08	14.69 ± 4.07
Canada	10.77 ± 3.77	11.27 ± 4.86	11.81 ± 5.97	10.40 ± 3.80	15.25 ± 4.45		15.17 ± 3.47	11.91 ± 3.81
Liechtenstein	17.17 ± 4.41	14.70 ± 4.35	17.54 ± 3.04	14.28 ± 3.91	15.47 ± 3.96	17.08 ± 3.03	13.58 ± 2.56	12.86 ± 6.57
USA	12.66 ± 4.14	13.53 ± 4.23	14.33 ± 4.11	13.08 ± 3.48	11.93 ± 3.60	12.98 ± 4.14	12.05 ± 4.83	13.13 ± 3.79
Belgium	14.31 ± 3.42	15.03 ± 4.37	15.70 ± 3.87	14.64 ± 4.89	15.56 ± 4.04	15.47 ± 4.08	14.05 ± 4.00	13.10 ± 3.38
Spain	15.98 ± 3.85	12.45 ± 4.53	15.46 ± 3.52	13.76 ± 4.25	15.59 ± 6.41	12.84 ± 3.40	15.76 ± 5.81	13.19 ± 3.71
Poland	13.60 ± 5.11	12.51 ± 2.25	10.35 ± 1.28	10.29 ± 0.05	13.84 ± 3.37	13.04 ± 4.68	10.45 ± 0.69	11.93

Data for Non-African runners are sorted in order of the number of finishers of each country

**Table 6:** Results of the mixed-effects regression analyses for change in running speed across years in female marathoners

Parameter	Estimate	SE	DF	T	*p* value	95 % CI	
						Upper	Lower
Ethiopia							
Constant term	147.793813	321.794557	36.803	0.459	0.649	−504.341821	799.929446
Year	−0.067200	0.160260	36.789	−0.419	0.677	−0.391981	0.257581
Cage	−0.041199	0.045279	39.863	−0.910	0.368	−0.132721	0.050323
Cage^2^	−0.000791	0.001556	27.290	−0.508	0.615	−0.003983	0.002401
Kenya							
Constant term	12.643951	134.413655	31.473	0.094	0.926	−261.327748	286.615651
Year	0.001310	0.067007	31.470	0.020	0.985	−0.135269	0.137890
Cage	0.004584	0.031623	33.197	0.145	0.886	−0.059740	0.068908
Cage^2^	0.000926	0.001504	28.885	0.616	0.543	−0.002150	0.004002
Austria							
Constant term	319.000059	151.126310	115.787	2.111	0.037	19.669554	618.330564
Year	−0.152749	0.075258	115.782	−2.030	0.045	−0.301810	−0.003688
Cage	−0.059867	0.033352	105.113	−1.795	0.076	−0.125998	0.006264
Cage^2^	0.003428	0.002369	91.358	1.447	0.151	−0.001277	0.008132
France							
Constant term	−69.668517	73.011026	423.181	−0.954	0.341	−213.177937	73.840903
Year	0.041647	0.036362	423.171	1.145	0.253	−0.029825	0.113119
Cage	−0.007736	0.019534	524.950	−0.396	0.692	−0.046111	0.030640
Cage^2^	−0.000529	0.001375	513.572	−0.385	0.701	−0.003230	0.002172
Great Britain							
Constant term	−32.852390	202.043235	96.505	−.163	0.871	−433.878261	368.173481
Year	0.023039	0.100666	96.499	0.229	0.819	−0.176769	0.222846
Cage	0.057327	0.032752	62.676	1.750	0.085	−0.008129	0.122782
Cage^2^	0.001266	0.003499	87.271	0.362	0.718	−0.005689	0.008220
Germany							
Constant term	−57.013601	61.704926	558.626	−0.924	0.356	−178.215628	64.188427
Year	0.035049	0.030733	558.613	1.140	0.255	−0.025317	0.095415
Cage	−0.018041	0.010289	360.049	−1.754	0.080	−0.038274	0.002192
Cage^2^	−0.000917	0.000669	425.048	−1.370	0.172	−0.002232	0.000399
Italy							
Constant term	12.643951	134.413655	31.473	0.094	0.926	−261.327748	286.615651
Year	0.001310	0.067007	31.470	0.020	0.985	−0.135269	0.137890
Cage	0.004584	0.031623	33.197	0.145	0.886	−0.059740	0.068908
Cage^2^	0.000926	0.001504	28.885	0.616	0.543	−0.002150	0.004002
Japan							
Constant term	−556.744907	324.548340	44.540	−1.715	0.093	−1210.605221	97.115408
Year	0.284663	0.161722	44.521	1.760	0.085	−0.041159	0.610486
Cage	0.002091	0.056456	36.733	0.037	0.971	−0.112328	0.116511
Cage^2^	−0.001140	0.002306	47.736	−.494	0.623	−0.005776	0.003496
Switzerland							
Constant term	19.166945	16.359822	5730.128	1.172	0.241	−12.904491	51.238380
Year	−0.001923	0.008148	5729.834	−0.236	0.813	−0.017896	0.014051
Cage	0.000169	0.002164	3789.103	0.078	0.938	−0.004074	0.004412
Cage^2^	−0.000271	0.000151	3903.532	−1.798	0.072	−0.000566	2.449756E−5
Canada							
Constant term	−84.838438	294.677013	21.509	−0.288	0.776	−696.771246	527.094370
Year	0.049412	0.146619	21.493	0.337	0.739	−0.255073	0.353897
Cage	−0.016467	0.105620	20.899	−0.156	0.878	−0.236180	0.203247
Cage^2^	−0.005069	0.008542	26.178	−0.593	0.558	−0.022621	0.012484
Principality of Liechtenstein							
Constant term	134.396574	114.930718	8.628	1.169	0.274	−127.312878	396.106027
Year	−0.058934	0.057365	8.641	−1.027	0.332	−0.189530	0.071661
Cage	0.000824	0.052738	11.742	0.016	0.988	−0.114363	0.116012
Cage^2^	−0.000182	0.005460	13.149	−0.033	0.974	−0.011963	0.011599
United States of America							
Constant term	147.793813	321.794557	36.803	0.459	0.649	−504.341821	799.929446
Year	−0.067200	0.160260	36.789	−0.419	0.677	−0.391981	0.257581
Cage	−0.041199	0.045279	39.863	−0.910	0.368	−0.132721	0.050323
Cage^2^	−0.000791	0.001556	27.290	−0.508	0.615	−0.003983	0.002401
Belgium							
Constant term	832.877872	481.304102	14.000	1.730	0.106	−199.416758	1865.172502
Year	−0.409025	0.239884	14.000	−1.705	0.110	−0.923525	0.105475
Cage	0.113307	0.073728	14.000	1.537	0.147	−0.044823	0.271437
Cage^2^	−0.008065	0.005950	14.000	−1.356	0.197	−0.020826	0.004695
Spain							
Constant term	578.599477	459.378710	18.000	1.260	0.224	−386.519821	1543.718775
Year	−0.280844	0.228570	18.000	−1.229	0.235	−0.761053	0.199365
Cage	0.085302	0.054009	9.306	1.579	0.148	−0.036264	0.206868
Cage^2^	−0.006105	0.004734	8.582	−1.290	0.231	−0.016894	0.004684
Poland							
Constant term	−1007.316625	429.834844	14.000	−2.343	0.034	−1929.220678	−85.412573
Year	0.507475	0.213730	14.000	2.374	0.032	0.049069	0.965881
Cage	−0.022253	0.077353	14.000	−0.288	0.778	−0.188158	0.143652
Cage^2^	0.001802	0.005561	14.000	0.324	0.751	−0.010126	0.013730

Data for Non-African runners are sorted in order of the number of finishers of each country

*Cage* centered age, *Cage* ^2^ centered age squared

**Table 7:** Results of the mixed-effects regression analyses for change in running speed across years in male marathoners

Parameter	Estimate	SE	DF	T	*p* value	95 % CI	
						Upper	Lower
Austria							
Constant term	119.014606	78.665485	352.571	1.513	0.131	−35.698004	273.727215
Year	−0.053124	0.039182	352.518	−1.356	0.176	−0.130183	0.023936
Cage	−0.053906	0.018537	374.501	−2.908	0.004	−0.090356	−0.017456
Cage^2^	0.002858	0.001180	374.482	2.423	0.016	0.000538	0.005179
France							
Constant term	12.224516	32.400764	1943.299	0.377	0.706	−51.319391	75.768423
Year	0.000774	0.016138	1943.341	0.048	0.962	−0.030876	0.032424
Cage	−0.042530	0.007858	2185.277	−5.412	0.000	−0.057940	−0.027119
Cage^2^	0.001220	0.000546	2369.390	2.235	0.025	0.000150	0.002290
Great Britain							
Constant term	168.008185	77.417245	265.471	2.170	0.031	15.578253	320.438116
Year	−0.076916	0.038558	265.527	−1.995	0.047	−0.152834	−0.000997
Cage	−0.003050	0.013447	228.557	−0.227	0.821	−0.029546	0.023446
Cage^2^	0.000290	0.001143	251.114	0.254	0.800	−0.001961	0.002541
Germany							
Constant term	−26.616836	25.282326	2510.280	−1.053	0.293	−76.193187	22.959515
Year	0.019883	0.012593	2510.229	1.579	0.114	−0.004811	0.044578
Cage	−0.007977	0.005083	2186.239	−1.570	0.117	−0.017944	0.001990
Cage^2^	0.000147	0.000344	2231.865	0.427	0.669	−0.000528	0.000822
Italy							
Constant term	82.322203	91.156734	288.896	0.903	0.367	−97.093338	261.737745
Year	−0.033932	0.045393	288.917	−0.748	0.455	−0.123275	0.055411
Cage	−0.058144	0.019311	261.042	−3.011	0.003	−0.096169	−0.020119
Cage^2^	0.000191	0.001214	322.202	0.157	0.875	−0.002199	0.002580
Japan							
Constant term	49.803604	170.811818	103.019	0.292	0.771	−288.960603	388.567811
Year	−0.018372	0.085088	103.003	−0.216	0.829	−0.187123	0.150380
Cage	−0.086555	0.031059	107.375	−2.787	0.006	−0.148123	−0.024987
Cage^2^	0.001926	0.001684	107.206	1.144	0.255	−0.001412	0.005264
Switzerland							
Constant term	25.878076	7.195438	22,373.993	3.596	0.000	11.774513	39.981639
Year	−0.005404	0.003584	22,373.571	−1.508	0.132	−0.012428	0.001620
Cage	−0.002664	0.001012	17,294.094	−2.633	0.008	−0.004647	−0.000681
Cage^2^	−0.000235	6.583543E−5	17,295.657	−3.565	0.000	−0.000364	−0.000106
Canada							
Constant term	−106.659105	171.420255	81.342	−0.622	0.536	−447.709874	234.391663
Year	0.059022	0.085420	81.336	0.691	0.492	−0.110927	0.228971
Cage	−0.050496	0.030360	68.401	−1.663	0.101	−0.111073	0.010080
Cage^2^	0.006306	0.001988	62.037	3.172	0.002	0.002332	0.010280
Principality of Liechtenstein							
Constant term	236.267620	241.570336	77.992	0.978	0.331	−244.662729	717.197969
Year	−0.109511	0.120377	77.993	−0.910	0.366	−0.349164	0.130142
Cage	0.034908	0.042003	73.075	0.831	0.409	−0.048802	0.118618
Cage^2^	0.001951	0.003222	72.523	0.606	0.547	−0.004471	0.008373
United States of America							
Constant term	179.260596	91.774367	188.890	1.953	0.052	−1.773750	360.294941
Year	−0.082585	0.045712	188.881	−1.807	0.072	−0.172757	0.007587
Cage	0.003829	0.014234	167.444	0.269	0.788	−0.024273	0.031931
Cage^2^	0.000319	0.000945	149.574	0.338	0.736	−0.001548	0.002186
Belgium							
Constant term	220.791210	167.798520	108.714	1.316	0.191	−111.789810	553.372230
Year	−0.102767	0.083486	108.675	−1.231	0.221	−0.268238	0.062704
Cage	−0.029095	0.030424	119.603	−0.956	0.341	−0.089335	0.031145
Cage^2^	0.000194	0.002163	96.384	0.090	0.929	−0.004099	0.004486
Spain							
Constant term	−130.890461	169.416654	5.528	−0.773	0.471	−554.170969	292.390047
Year	0.072247	0.084311	5.528	0.857	0.427	−0.138405	0.282899
Cage	−0.050933	0.030883	5.028	−1.649	0.160	−0.130185	0.028319
Cage^2^	0.001306	0.004902	56.210	0.266	0.791	−0.008513	0.011125
Poland							
Constant term	−173.031933	285.748419	50.731	−0.606	0.548	−746.769620	400.705753
Year	0.092292	0.142290	50.736	0.649	0.520	−0.193403	0.377986
Cage	−0.019397	0.026881	30.284	−0.722	0.476	−0.074273	0.035480
Cage^2^	−0.000780	0.001881	35.444	−0.414	0.681	−0.004597	0.003038
Kenya							
Constant term	31.727578	85.570347	33.000	0.371	0.713	−142.366603	205.821759
Year	−0.007558	0.042603	33.000	−0.177	0.860	−0.094234	0.079119
Cage	−0.192695	0.042007	33.000	−4.587	0.000	−0.278159	−0.107230
Cage^2^	−0.005855	0.002387	33.000	−2.453	0.020	−0.010712	−0.000999
Ethiopia							
Constant term	185.271970	155.298262	15.000	1.193	0.251	−145.738439	516.282379
Year	−0.085404	0.077000	15.000	−1.109	0.285	−0.249526	0.078718
Cage	−0.708418	0.358618	15.000	−1.975	0.067	−1.472793	0.055957
Cage^2^	−0.034788	0.014889	15.000	−2.337	0.034	−0.066522	−0.003053

Data for Non-African runners are sorted in order of the number of finishers of each country

*Cage* centered age, *Cage* ^2^ centered age squared

**Table 8:** Age (years) with mean ± SD of female and male East-African and Non-African half-marathoners

	1999	2000	2001	2002	2003	2004	2005	2006
Women								
Ethiopia	20	26 ± 1	30		36	37	35	26 ± 10
Kenya		29 ± 9		35 ± 6	37 ± 1	29 ± 6	28 ± 7	28 ± 5
Austria		40	50 ± 8	44 ± 3	37 ± 8		38 ± 7	44 ± 1
Canada		49 ± 4	44 ± 10	40 ± 9	44 ± 9	53 ± 7	41 ± 13	35 ± 6
Czech Republic	37 ± 14	49	31 ± 5	33 ± 18	34 ± 7	34 ± 5	35 ± 5	27
Denmark		36 ± 0	34 ± 4	36 ± 5	33 ± 9	42 ± 12	48 ± 9	46 ± 7
Spain	43 ± 22	40 ± 16	36 ± 5	37 ± 7	38 ± 8	37 ± 9	43 ± 9	37 ± 9
France	42 ± 10	43 ± 10	41 ± 9	41 ± 9	42 ± 9	42 ± 10	42 ± 10	42 ± 10
Great Britain	37 ± 7	39 ± 10	41 ± 12	39 ± 9	39 ± 8	36 ± 8	38 ± 9	39 ± 10
Germany	43 ± 10	43 ± 9	45 ± 10	43 ± 9	44 ± 9	44 ± 9	43 ± 9	43 ± 10
Italy	41 ± 9	48 ± 9	46 ± 11	41 ± 9	42 ± 11	42 ± 9	42 ± 10	39 ± 9
Japan	36 ± 13	57 ± 22	61 ± 7	50 ± 15	50 ± 12	54 ± 12	49 ± 15	48 ± 18
Liechtenstein	44 ± 4	46 ± 10	41 ± 6	45 ± 8	40 ± 8	39 ± 9	40 ± 8	44 ± 10
Luxembourg	47 ± 21	42 ± 11	35 ± 7	40 ± 7	44 ± 12	42 ± 14	37 ± 8	45 ± 7
Netherlands	44 ± 6	47 ± 1	43 ± 13	40 ± 7	43 ± 11	45 ± 11	40 ± 11	43 ± 10
Norway		60 ± 1	55 ± 16	39 ± 16	56	41 ± 13	45 ± 15	50 ± 13
Portugal		54	41	44 ± 14	46 ± 9	36 ± 9	38 ± 10	36 ± 8
Switzerland	41 ± 10	41 ± 10	41 ± 10	41 ± 10	41 ± 10	41 ± 10	41 ± 10	41 ± 10
USA	26 ± 3	35 ± 9	36 ± 10	44 ± 19	34 ± 12	36 ± 10	42 ± 12	41 ± 13
Australia		40	50 ± 8	44 ± 3	37 ± 8		38 ± 7	44 ± 1
Belgium	53 ± 21		47 ± 21	38 ± 16	44 ± 10	37 ± 10	38 ± 11	44 ± 7
Hungary	68	65		72	43	41	33	46 ± 7
Ireland	37 ± 7	44	38 ± 1	43 ± 2	42 ± 11	36 ± 6		41 ± 5
Poland	44 ± 12		43 ± 7	30	36 ± 11	39 ± 10	45 ± 11	34 ± 5
Russia	52	29 ± 5	28	30		42 ± 11	38 ± 12	32 ± 5
Sweden	27		42 ± 12	48 ± 11	34 ± 7	38 ± 6	43 ± 16	44 ± 13
Finland	33 ± 1	44		44 ± 18		40 ± 10	44 ± 12	40 ± 4
Greece					39	48	32	33
South Africa				47 ± 11	35	52	36	44 ± 1
Brazil	45 ± 16	50 ± 4		40	41	46	50 ± 4	
Mexico						37		38 ± 6
Argentina				38				32
India		29				47	36 ± 7	43 ± 11
Israel						64	59	
Slovenia							47	
Men								
Ethiopia	30 ± 6	27	30 ± 3	27 ± 4	23 ± 1	24 ± 2	25 ± 6	28 ± 3
Kenya	26 ± 1	26 ± 5	30 ± 3	29 ± 5	32 ± 2	37 ± 20	32 ± 18	27 ± 5
Austria		43 ± 5	38 ± 5	38 ± 13	40 ± 11	42 ± 12	35 ± 7	41 ± 6
Canada	40 ± 10	39 ± 12	40 ± 16	36 ± 11	39 ± 12	40 ± 10	42 ± 14	37 ± 10
Czech Republic	37 ± 6	37 ± 13	31 ± 10	33 ± 6	40 ± 13	38 ± 8	35 ± 9	37 ± 11
Denmark		47 ± 8	47 ± 14	41 ± 12	33 ± 9	47 ± 14	40 ± 9	43 ± 14
Spain	35 ± 11	42 ± 8	34 ± 7	42 ± 10	42 ± 11	43 ± 10	43 ± 11	42 ± 10
France	41 ± 10	42 ± 10	41 ± 9	42 ± 10	42 ± 10	41 ± 10	42 ± 10	41 ± 9
Great Britain	40 ± 10	40 ± 10	41 ± 11	39 ± 10	41 ± 11	39 ± 10	41 ± 10	43 ± 11
Germany	44 ± 10	44 ± 10	43 ± 10	43 ± 10	43 ± 10	43 ± 10	43 ± 10	43 ± 10
Italy	44 ± 11	42 ± 8	43 ± 10	42 ± 10	43 ± 9	41 ± 9	42 ± 8	41 ± 9
Japan	66 ± 5	51 ± 16	48 ± 13	54 ± 11	47 ± 19	46 ± 15	55 ± 14	44 ± 17
Liechtenstein	38 ± 7	40 ± 8	44 ± 9	41 ± 11	40 ± 9	41 ± 9	41 ± 11	41 ± 10
Luxembourg	43 ± 14	37 ± 3	37 ± 6	43 ± 11	43 ± 10	43 ± 9	42 ± 9	42 ± 11
Netherlands	50 ± 10	41 ± 16	35 ± 9	39 ± 11	36 ± 8	38 ± 12	38 ± 7	39 ± 10
Norway	33	28 ± 6	43 ± 14	40 ± 13	39 ± 8	32 ± 9	34 ± 7	44 ± 19
Portugal	45 ± 8	46 ± 8	41 ± 9	38 ± 9	39 ± 11	37 ± 8	38 ± 6	39 ± 10
Switzerland	41 ± 10	41 ± 10	41 ± 10	41 ± 10	41 ± 10	41 ± 10	41 ± 10	41 ± 10
USA	41 ± 12	43 ± 12	39 ± 11	41 ± 11	41 ± 13	41 ± 10	40 ± 9	40 ± 12
Australia		43 ± 5	38 ± 5	38 ± 13	40 ± 11	42 ± 12	35 ± 7	41 ± 6
Belgium	43 ± 16	44 ± 11	43 ± 9	42 ± 10	40 ± 11	36 ± 10	42 ± 13	42 ± 11
Hungary		41	46 ± 16	39 ± 10	48 ± 15	47 ± 14	42 ± 16	43 ± 13
Ireland	34 ± 10	38 ± 6	42 ± 9	42 ± 7	40 ± 11	37 ± 5	35 ± 4	37 ± 6
Poland	40 ± 12	38 ± 8	40 ± 7	37 ± 9	32 ± 16	37 ± 17	38 ± 13	39 ± 11
Russia		38 ± 9	34 ± 11	33 ± 8	40 ± 15	41 ± 7	40 ± 12	34 ± 6
Sweden	47 ± 13	45 ± 19	40 ± 8	43 ± 10	46 ± 14	40 ± 13	42 ± 11	43 ± 12
Finland	61 ± 8	41 ± 10	47 ± 13	40 ± 10	44 ± 16	43 ± 13	43 ± 11	42 ± 13
Greece	37	31	43 ± 4	31 ± 9	34 ± 7	37 ± 6	44 ± 13	33 ± 4
South Africa	40			52		39 ± 6	38	41 ± 17
Brazil	46 ± 18	53 ± 13	44 ± 14		42 ± 11			47 ± 6
Mexico	46	40	52	38 ± 13	40 ± 8	47 ± 13	46	30 ± 10
Argentina					39	30	51	39
India		40 ± 9	48 ± 2	34	42 ± 7		38 ± 9	36 ± 8
Israel		46 ± 6		50 ± 4	36 ± 2	31 ± 4	39	43 ± 26
Slovenia		62	35	48 ± 6	39	44	39 ± 3	37 ± 2

	2007	2008	2009	2010	2011	2012	2013	2014
Women								
Ethiopia	29 ± 5	41 ± 14	34 ± 8	28 ± 6	29 ± 7	27 ± 7	33 ± 8	27 ± 5
Kenya	27 ± 6	29 ± 5	29 ± 8	29 ± 0	34 ± 9	30 ± 4	30 ± 6	29 ± 4
Austria	53 ± 12	35	41 ± 2	40 ± 11	55 ± 19	48 ± 14	43 ± 5	40 ± 5
Canada	37 ± 7	37 ± 8	35 ± 7	37 ± 10	43 ± 11	41 ± 11	38 ± 11	41 ± 13
Czech Republic	40 ± 9	42 ± 8	40 ± 8	32 ± 4	34 ± 8	38 ± 9	34 ± 11	33 ± 6
Denmark	37 ± 6	45	46 ± 7	41 ± 12	36 ± 12	40 ± 9	35 ± 4	43 ± 9
Spain	42 ± 10	43 ± 8	41 ± 9	43 ± 5	44 ± 7	38 ± 9	43 ± 10	43 ± 10
France	43 ± 10	41 ± 9	42 ± 9	42 ± 9	41 ± 9	40 ± 10	41 ± 10	41 ± 9
Great Britain	38 ± 11	38 ± 9	38 ± 9	41 ± 11	39 ± 10	41 ± 10	39 ± 9	38 ± 10
Germany	43 ± 10	43 ± 10	44 ± 10	43 ± 10	43 ± 10	44 ± 10	43 ± 10	43 ± 10
Italy	42 ± 11	43 ± 10	41 ± 9	44 ± 10	42 ± 10	43 ± 10	42 ± 9	42 ± 9
Japan	50 ± 17	45 ± 16	42 ± 14	42 ± 13	52 ± 14	53 ± 13	50 ± 9	47 ± 16
Liechtenstein	42 ± 12	38 ± 10	40 ± 10	41 ± 10	39 ± 10	46 ± 10	43 ± 9	38 ± 9
Luxembourg	41 ± 9	46 ± 12	38 ± 7	37 ± 9	37 ± 7	43 ± 9	41 ± 10	44 ± 12
Netherlands	43 ± 9	46 ± 9	48 ± 10	43 ± 8	42 ± 9	50 ± 13	41 ± 8	46 ± 11
Norway	47 ± 13	52 ± 13	56 ± 15	36 ± 14	41 ± 13	33 ± 6	49 ± 19	63 ± 2
Portugal	42 ± 10	45 ± 11	48 ± 6	46 ± 5	36	41 ± 13	40 ± 4	46 ± 4
Switzerland	41 ± 10	41 ± 10	41 ± 10	41 ± 10	41 ± 10	41 ± 10	41 ± 10	41 ± 10
USA	38 ± 11	38 ± 10	38 ± 8	38 ± 11	42 ± 13	37 ± 9	38 ± 10	39 ± 10
Australia	53 ± 12	35	41 ± 2	40 ± 11	55 ± 19	48 ± 14	43 ± 5	40 ± 5
Belgium	43 ± 14	47 ± 12	39 ± 11	42 ± 10	43 ± 11	42 ± 6	44 ± 8	37 ± 8
Hungary	48 ± 10	41	47 ± 9	47	47 ± 18	42 ± 8	51 ± 14	50 ± 12
Ireland	32	49	33 ± 5	36 ± 10	32 ± 5	43 ± 4	40 ± 7	38 ± 11
Poland	38 ± 6	33 ± 5	32 ± 7	45 ± 13	43 ± 10	44 ± 13	37 ± 9	43 ± 10
Russia	38	33 ± 5	36 ± 8	34 ± 14	34 ± 6	34 ± 11	37 ± 16	36 ± 7
Sweden	58 ± 11	44 ± 13	40 ± 14	37 ± 10	44 ± 12	41 ± 9	42 ± 13	41 ± 10
Finland	48 ± 5	57 ± 2	48 ± 15	41 ± 9	54 ± 7	43 ± 10	47 ± 11	44 ± 8
Greece	33 ± 1	45	47	40	33	34 ± 6	53 ± 14	40 ± 4
South Africa	37 ± 7	48 ± 15	40 ± 5		52 ± 13	47 ± 14	39 ± 9	47
Brazil	52 ± 26	45 ± 16	40 ± 15	41 ± 6	45 ± 7	43 ± 19	43 ± 9	39 ± 5
Mexico	36 ± 2	43 ± 21	43 ± 5	41 ± 11	50 ± 5	39 ± 9	43	38 ± 5
Argentina	36		28	43 ± 4	34	46 ± 11	40 ± 10	36 ± 2
India	41 ± 15		25		35 ± 6		33 ± 5	42 ± 1
Israel			28	31	52 ± 0	29	36 ± 11	44 ± 2
Slovenia	41		47 ± 9		30 ± 4			42
Men								
Ethiopia	31 ± 5	30 ± 6	25 ± 6	27 ± 4	32 ± 7	33 ± 7	26 ± 5	27 ± 5
Kenya	29 ± 4	27 ± 4	32 ± 13	30 ± 5	29 ± 7	29 ± 5	28 ± 4	31 ± 5
Austria	40 ± 13	35 ± 6	35 ± 9	43 ± 11	36 ± 8	44 ± 15	41 ± 11	39 ± 8
Canada	37 ± 13	36 ± 14	36 ± 10	41 ± 11	37 ± 14	42 ± 12	40 ± 10	39 ± 12
Czech Republic	39 ± 12	41 ± 13	40 ± 12	37 ± 11	38 ± 13	36 ± 8	35 ± 10	41 ± 10
Denmark	40 ± 11	40 ± 10	42 ± 7	45 ± 12	40 ± 10	44 ± 9	39 ± 10	43 ± 10
Spain	42 ± 9	41 ± 11	40 ± 8	40 ± 10	41 ± 10	41 ± 9	40 ± 10	36 ± 8
France	42 ± 10	42 ± 10	41 ± 10	42 ± 10	42 ± 10	42 ± 9	42 ± 9	42 ± 10
Great Britain	41 ± 11	40 ± 11	41 ± 11	40 ± 10	41 ± 10	40 ± 10	40 ± 10	40 ± 11
Germany	43 ± 10	43 ± 10	43 ± 10	43 ± 10	43 ± 10	44 ± 10	43 ± 10	43 ± 10
Italy	42 ± 10	43 ± 9	43 ± 10	43 ± 10	43 ± 10	43 ± 9	43 ± 10	44 ± 10
Japan	47 ± 17	49 ± 18	49 ± 16	46 ± 17	52 ± 16	50 ± 15	54 ± 15	48 ± 14
Liechtenstein	42 ± 10	42 ± 9	42 ± 9	41 ± 8	43 ± 9	41 ± 9	41 ± 9	41 ± 9
Luxembourg	41 ± 10	38 ± 8	38 ± 12	37 ± 10	39 ± 11	39 ± 6	46 ± 7	46 ± 9
Netherlands	44 ± 12	45 ± 10	43 ± 11	42 ± 11	44 ± 10	42 ± 11	42 ± 11	43 ± 9
Norway	45 ± 11	37 ± 11	44 ± 11	51 ± 14	41 ± 15	45 ± 10	51 ± 11	43 ± 12
Portugal	38 ± 9	39 ± 10	42 ± 8	36 ± 9	39 ± 11	38 ± 8	42 ± 12	44 ± 8
Switzerland	41 ± 10	41 ± 10	41 ± 10	41 ± 10	41 ± 10	41 ± 10	41 ± 10	41 ± 10
USA	38 ± 11	42 ± 11	39 ± 10	40 ± 9	38 ± 11	40 ± 12	39 ± 10	41 ± 10
Australia	40 ± 13	35 ± 6	35 ± 9	43 ± 11	36 ± 8	44 ± 15	41 ± 11	39 ± 8
Belgium	40 ± 11	39 ± 8	39 ± 10	42 ± 12	42 ± 10	44 ± 11	43 ± 10	44 ± 11
Hungary	43 ± 14	43 ± 9	52 ± 16	45 ± 12	42 ± 15	46 ± 16	38 ± 15	47 ± 9
Ireland	39 ± 9	45 ± 12	37 ± 8	39 ± 11	42 ± 9	41 ± 11	44 ± 11	43 ± 9
Poland	40 ± 9	40 ± 13	38 ± 14	39 ± 13	39 ± 11	38 ± 11	38 ± 10	37 ± 11
Russia	33 ± 6	42 ± 7	37 ± 10	35 ± 10	35 ± 7	37 ± 10	40 ± 12	36 ± 6
Sweden	45 ± 9	41 ± 11	44 ± 12	42 ± 14	46 ± 12	45 ± 12	43 ± 13	43 ± 12
Finland	41 ± 13	41 ± 8	46 ± 14	41 ± 9	43 ± 13	43 ± 12	40 ± 12	41 ± 11
Greece	37 ± 8	43 ± 11	44 ± 14	43 ± 14	33 ± 7	52 ± 10	39 ± 11	44 ± 14
South Africa	41 ± 9	30 ± 5	30 ± 5	44 ± 7	34 ± 4	37 ± 16	39 ± 9	38 ± 8
Brazil	47 ± 10	43 ± 7	41 ± 7	45 ± 13	44 ± 11	44 ± 11	44 ± 8	47 ± 11
Mexico	45 ± 12	36 ± 9	47 ± 3	41 ± 4	41 ± 8	40 ± 14	40 ± 7	41 ± 7
Argentina	44	46	35 ± 4	38 ± 4	40 ± 5	42 ± 9	36 ± 5	41 ± 7
India	38	33 ± 9	46 ± 11	42 ± 14	39 ± 6	32 ± 6	34 ± 7	37 ± 5
Israel	39 ± 12	39 ± 12	42 ± 10	47 ± 17	43 ± 12	47 ± 15	41 ± 9	41 ± 10
Slovenia			42 ± 21	41	39	43 ± 16	39 ± 11	

Data for Non-African runners are sorted in order of the number of finishers of each country

**Table 9:** Results of the mixed-effects regression analyses for change in age across years in half-marathoners

Parameter	Estimate	SE	DF	T	*p* value
Ethiopia					
Constant term	−182.511370	314.486675	91.467	−0.580	0.563
Female sex	1.680182	1.432541	72.982	1.173	0.245
Calendar year	0.104919	0.156633	91.480	0.670	0.505
Kenya					
Constant term	8.235275	288.742638	208.395	0.029	0.977
Female sex	−0.121674	1.317439	68.640	−0.092	0.927
Calendar year	0.010794	0.143794	208.376	0.075	0.940
Austria					
Constant term	194.074686	73.655324	2996.146	2.635	0.008
Female sex	−1.709831	0.481769	1362.215	−3.549	<0.0001
Calendar year	−0.075364	0.036673	2996.074	−2.055	0.040
Canada					
Constant term	160.945071	202.099830	612.252	0.796	0.426
Female sex	−1.870259	1.338345	281.258	−1.397	0.163
Calendar year	−0.059563	0.100658	612.236	−0.592	0.554
Czech Republic					
Constant term	−103.672165	314.871371	203.897	−0.329	0.742
Female sex	−1.866522	1.674310	123.397	−1.115	0.267
Calendar year	0.070316	0.156835	203.856	0.448	0.654
Denmark					
Constant term	635.928991	345.802767	242.013	1.839	0.067
Female sex	−1.207578	1.995708	124.433	−0.605	0.546
Calendar year	−0.295499	0.172164	242.020	−1.716	0.087
Spain					
Constant term	4.345138	173.122867	707.441	0.025	0.980
Female sex	−0.414857	0.961543	416.419	−0.431	0.666
Calendar year	0.018514	0.086181	707.444	0.215	0.830
France					
Constant term	40.876029	30.739542	20,221.009	1.330	0.184
Female sex	−0.363674	0.201893	9121.960	−1.801	0.072
Calendar year	0.000558	0.015305	20,220.731	0.036	0.971
Great Britain					
Constant term	62.808682	86.098036	2964.716	0.730	0.466
Female sex	−1.582820	0.555386	1329.593	−2.850	0.004
Calendar year	−0.010529	0.042874	2964.669	−0.246	0.806
Germany					
Constant term	47.516840	32.418394	21,887.424	1.466	0.143
Female sex	0.269587	0.192793	9869.455	1.398	0.162
Calendar year	−0.002199	0.016141	21,888.087	−0.136	0.892
Italy					
Constant term	99.335607	63.935535	3599.246	1.554	0.120
Female sex	−0.964511	0.478133	1789.232	−2.017	0.044
Calendar year	−0.027856	0.031834	3599.102	−0.875	0.382
Japan					
Constant term	774.526324	242.816752	456.029	3.190	0.002
Female sex	−0.297851	1.873820	298.110	−0.159	0.874
Calendar year	−0.361601	0.120894	456.006	−2.991	0.003
Liechtenstein					
Constant term	86.887276	148.175736	975.830	0.586	0.558
Female sex	−0.191866	0.758997	586.133	−0.253	0.801
Calendar year	−0.022614	0.073778	975.840	−0.307	0.759
Luxembourg					
Constant term	−164.038413	297.385871	212.919	−0.552	0.582
Female sex	−0.327141	1.355426	151.704	−0.241	0.810
Calendar year	0.102236	0.148098	212.984	0.690	0.491
Netherlands					
Constant term	−523.086955	207.948527	597.809	−2.515	0.012
Female sex	0.885014	1.109896	324.076	0.797	0.426
Calendar year	0.281968	0.103524	597.796	2.724	0.007
Norway					
Constant term	−912.882656	459.239160	164.453	−1.988	0.048
Female sex	6.870747	2.584284	91.195	2.659	0.009
Calendar year	0.475098	0.228639	164.454	2.078	0.039
Portugal					
Constant term	−51.331697	261.757709	235.116	−0.196	0.845
Female sex	1.523814	1.797020	119.389	0.848	0.398
Calendar year	0.046577	0.130365	235.105	0.357	0.721
Switzerland					
Constant term	41.194097	0.025301	124,980.522	1.174	0.101
Female sex	0.130365	0.045674	144,203.009	2.854	0.401
Calendar year	−0.003405	0.004187		−0.813	0.759
United States of America					
Constant term	59.833065	149.760218	1239.031	0.400	0.690
Female sex	−0.210582	0.907549	602.658	−0.232	0.817
Calendar year	−0.009517	0.074571	1239.035	−0.128	0.898
Australia					
Constant term	352.823540	240.345850	102.968	1.468	0.145
Female sex	0.724548	1.955842	120.491	0.370	0.712
Calendar year	−0.155166	0.119654	102.965	−1.297	0.198
Belgium					
Constant term	32.115986	200.805249	711.224	0.160	0.873
Female sex	0.194925	1.089004	423.817	0.179	0.858
Calendar year	0.005088	0.099966	711.329	0.051	0.959
Hungary					
Constant term	306.588165	391.814434	209.053	0.782	0.435
Female sex	1.793288	2.174815	181.126	0.825	0.411
Calendar year	−0.129388	0.195061	209.050	−0.663	0.508
Ireland					
Constant term	−234.096789	266.979012	233.252	−0.877	0.381
Female sex	−1.746638	1.660739	110.543	−1.052	0.295
Calendar year	0.136751	0.132924	233.250	1.029	0.305
Poland					
Constant term	80.619356	304.876109	293.818	0.264	0.792
Female sex	1.372858	1.636758	169.363	0.839	0.403
Calendar year	−0.020831	0.151805	293.811	−0.137	0.891
Russia					
Constant term	172.423406	334.325523	166.780	0.516	0.607
Female sex	−1.652883	1.736428	115.009	−0.952	0.343
Calendar year	−0.067115	0.166487	166.786	−0.403	0.687
Sweden					
Constant term	289.316852	335.082052	353.960	0.863	0.388
Female sex	−0.592306	1.563797	209.865	−0.379	0.705
Calendar year	−0.122190	0.166817	353.950	−0.732	0.464
Finland					
Constant term	−59.142933	311.996766	307.703	−0.190	0.850
Female sex	3.608451	1.778953	155.630	2.028	0.044
Calendar year	0.049886	0.155324	307.703	0.321	0.748
Greece					
Constant term	−892.527239	519.331793	93.956	−1.719	0.089
Female sex	−0.405076	2.897113	68.342	−0.140	0.889
Calendar year	0.464430	0.258636	93.954	1.796	0.076
Republic of South Africa					
Constant term	636.290049	640.320419	75.353	0.994	0.324
Female sex	2.142126	2.784791	44.423	0.769	0.446
Calendar year	−0.296861	0.318625	75.353	−0.932	0.354
Brazil					
Constant term	390.737458	494.198576	122.510	0.791	0.431
Female sex	−1.389051	2.469821	73.141	−0.562	0.576
Calendar year	−0.171769	0.245978	122.514	−0.698	0.486
Mexico					
Constant term	342.919947	496.145544	92.343	0.691	0.491
Female sex	−0.070202	2.152566	68.580	−0.033	0.974
Calendar year	−0.150281	0.246976	92.326	−0.608	0.544
Argentina					
Constant term	−133.127127	591.831333	31.764	−0.225	0.823
Female sex	−2.442123	2.570733	24.433	−0.950	0.351
Calendar year	0.086027	0.294531	31.768	0.292	0.772
India					
Constant term	647.670352	567.767634	57.387	1.141	0.259
Female sex	−0.840896	2.566219	39.798	−0.328	0.745
Calendar year	−0.302860	0.282792	57.372	−1.071	0.289
Israel					
Constant term	369.720687	818.353267	61.363	0.452	0.653
Female sex	2.219258	4.361460	43.335	0.509	0.613
Calendar year	−0.162596	0.407397	61.348	−0.399	0.691
Slovenia					
Constant term	−780.162274	551.609835	27.991	−1.414	0.168
Female sex	−7.106008	2.764787	23.422	−2.570	0.017
Calendar year	0.409617	0.274536	27.999	1.492	0.147

Data for Non-African runners are sorted in order of the number of finishers of each country

**Table 10:** Age (years) with mean ± SD of female and male East-African and Non-African marathoners

	1999	2000	2001	2002	2003	2004	2005	2006
Women								
Ethiopia							32	
Kenya						32		
Austria	47 ± 4	45 ± 15	32	26 ± 8	37 ± 4	45 ± 7	40 ± 11	41 ± 11
France	40 ± 9	47 ± 7	45 ± 11	43 ± 9	47 ± 10	46 ± 9	45 ± 8	44 ± 8
Great Britain		34 ± 4	42 ± 18	29 ± 12	43 ± 11	40 ± 9	40 ± 9	40 ± 12
Germany	45 ± 9	46 ± 11	48 ± 12	48 ± 11	44 ± 10	45 ± 13	44 ± 10	44 ± 9
Italy	43	61 ± 16	36 ± 16	52 ± 2	50 ± 4	48 ± 8	40 ± 6	33 ± 4
Japan	63	66	42 ± 17	43 ± 30	57	47 ± 18	53 ± 17	52
Switzerland	41 ± 11	42 ± 10	42 ± 11	41 ± 10	42 ± 10	42 ± 11	43 ± 11	41 ± 11
Canada				38	49 ± 10	55 ± 1	54	48 ± 7
Liechtenstein		44	52 ± 8		42 ± 11		48 ± 21	40 ± 4
USA		51				29 ± 2	39 ± 14	40 ± 17
Belgium	28				41	43 ± 18	41 ± 13	46 ± 13
Spain							40	
Poland				25	30 ± 1			
Men								
Ethiopia			28 ± 3					
Kenya		33	24 ± 4		29 ± 6	29 ± 8	29	
Austria	52 ± 4	43 ± 14	45 ± 10	45 ± 9	42 ± 7	45 ± 7	44 ± 8	44 ± 9
France	43 ± 8	41 ± 10	44 ± 11	44 ± 10	44 ± 10	44 ± 9	44 ± 9	43 ± 9
Great Britain	34 ± 8	38 ± 16	39 ± 11	46 ± 13	43 ± 12	39 ± 10	41 ± 11	43 ± 10
Germany	40 ± 9	44 ± 9	44 ± 9	45 ± 9	44 ± 9	44 ± 9	43 ± 9	43 ± 9
Italy	52 ± 10	45 ± 8	50 ± 8	42 ± 9	44 ± 9	43 ± 12	44 ± 13	41 ± 9
Japan	41	64	36 ± 7	64	57 ± 8	51 ± 13	46 ± 17	45 ± 14
Switzerland	42 ± 11	43 ± 11	42 ± 11	42 ± 11	42 ± 11	42 ± 10	42 ± 11	42 ± 11
Canada	44 ± 6	45	33 ± 6	44 ± 5	41 ± 12	38 ± 12	43 ± 14	42 ± 16
Liechtenstein			29	53 ± 15	44 ± 10	40 ± 8	41 ± 8	43 ± 7
USA	45 ± 9	55 ± 8	41 ± 8	36 ± 14	37 ± 10	40 ± 11	44 ± 10	42 ± 13
Belgium		38		44 ± 10	41 ± 12	37 ± 15	43 ± 18	43 ± 11
Spain		54	45	28 ± 3	57 ± 9	44 ± 4	42 ± 10	41 ± 11
Poland		31		27 ± 4	58	40 ± 12	49	30 ± 1

	2007	2008	2009	2010	2011	2012	2013	2014
Women								
Ethiopia					21	26		
Kenya					35			
Austria	44 ± 7	39 ± 10	43 ± 7	43 ± 6	44 ± 4	45 ± 7	40 ± 3	41 ± 10
France	41 ± 9	44 ± 9	42 ± 10	43 ± 10	45 ± 9	43 ± 8	43 ± 10	45 ± 9
Great Britain	40 ± 10	37 ± 9	39 ± 10	38 ± 18	38 ± 7	45 ± 9	36 ± 8	40 ± 12
Germany	43 ± 8	44 ± 10	43 ± 10	41 ± 11	44 ± 11	44 ± 10	41 ± 11	45 ± 10
Italy	44 ± 13	44 ± 14	55 ± 16	45 ± 10	41 ± 19	55 ± 11	41 ± 23	44 ± 18
Japan	54 ± 18	50 ± 18	61	50 ± 19	56 ± 13	49 ± 11	56 ± 8	57 ± 2
Switzerland	42 ± 10	43 ± 11	42 ± 11	41 ± 11	42 ± 11	42 ± 10	42 ± 11	42 ± 11
Canada	25	41 ± 17	41 ± 4	38 ± 10	41 ± 15	32 ± 0	35 ± 3	33 ± 7
Liechtenstein	33 ± 10	33 ± 8	39 ± 9	42	53	39 ± 8		46 ± 21
USA	44 ± 17	44 ± 22	37 ± 11	62 ± 8	50 ± 25	31		41 ± 20
Belgium	52 ± 13	29		46	54			
Spain	46 ± 8	34	43 ± 7		41 ± 2	43 ± 10	44	40 ± 13
Poland			18	38	52 ± 1	49 ± 13	40 ± 11	39 ± 2
Men								
Ethiopia	24 ± 4	26	32 ± 1	23 ± 8	32		28 ± 8	26
Kenya	35 ± 13	28 ± 1	28 ± 3	32 ± 5	29 ± 6	24	27 ± 6	30 ± 2
Austria	41 ± 10	42 ± 11	44 ± 10	43 ± 6	42 ± 8	42 ± 8	42 ± 7	45 ± 9
France	43 ± 9	44 ± 10	42 ± 11	43 ± 10	44 ± 9	42 ± 10	42 ± 10	44 ± 10
Great Britain	41 ± 8	41 ± 11	39 ± 11	41 ± 9	41 ± 9	42 ± 10	41 ± 11	35 ± 8
Germany	44 ± 10	43 ± 9	44 ± 10	43 ± 10	43 ± 10	43 ± 10	44 ± 10	44 ± 10
Italy	41 ± 8	42 ± 8	41 ± 9	47 ± 9	45 ± 12	43 ± 8	46 ± 9	43 ± 12
Japan	48 ± 14	48 ± 14	48 ± 19	49 ± 17	49 ± 17	42 ± 16	46 ± 19	64 ± 7
Switzerland	42 ± 11	42 ± 11	42 ± 11	42 ± 10	42 ± 11	42 ± 10	42 ± 11	42 ± 10
Canada	35 ± 12	42 ± 9	49 ± 8	46 ± 14	41 ± 9		40 ± 7	50 ± 9
Liechtenstein	36 ± 14	42 ± 10	43 ± 5	42 ± 7	37 ± 4	30 ± 4	31 ± 1	42 ± 13
USA	45 ± 10	39 ± 10	41 ± 12	40 ± 9	41 ± 9	36 ± 7	41 ± 13	42 ± 10
Belgium	39 ± 8	38 ± 10	43 ± 11	44 ± 10	48 ± 13	42 ± 12	44 ± 10	42 ± 11
Spain	42 ± 10	47 ± 11	41 ± 9	35 ± 12	40 ± 7	33 ± 6	40 ± 12	44 ± 6
Poland	47 ± 5	38 ± 8	42 ± 14	32 ± 9	38 ± 14	50 ± 16	35 ± 15	56

Data for Non-African runners are sorted in order of the number of finishers of each country

**Table 11:** Results of the mixed-effects regression analyses for change in age across years in marathoners

Parameter	Estimate	SE	DF	T	*p* value
Ethiopia					
Constant term	129.282320	508.886678	16.700	0.254	0.803
Female sex	−0.243651	3.310608	12.319	−0.074	0.943
Calendar year	−0.050632	0.253416	16.702	−0.200	0.844
Kenya					
Constant term	−112.654090	528.060345	30.357	−0.213	0.832
Female sex	3.707084	4.569460	13.457	0.811	0.431
Calendar year	0.070957	0.263161	30.291	0.270	0.789
Austria					
Constant term	203.368427	202.655612	491.365	1.004	0.316
Female sex	−2.565643	1.122330	309.176	−2.286	0.023
Calendar year	−0.079728	0.100942	491.362	−0.790	0.430
France					
Constant term	118.121641	83.994410	2805.626	1.406	0.160
Female sex	0.044112	0.552283	1850.356	0.080	0.936
Calendar year	−0.037087	0.041835	2805.619	−0.887	0.375
Great Britain					
Constant term	389.692562	249.759353	477.933	1.560	0.119
Female sex	−1.714608	1.280914	348.174	−1.339	0.182
Calendar year	−0.173769	0.124396	477.931	−1.397	0.163
Germany					
Constant term	107.789331	82.298718	3899.573	1.310	0.190
Female sex	0.535367	0.472119	2458.948	1.134	0.257
Calendar year	−0.032069	0.040991	3899.516	−0.782	0.434
Italy					
Constant term	556.010922	261.000704	423.965	2.130	0.034
Female sex	1.394311	1.647994	289.562	0.846	0.398
Calendar year	−0.255065	0.129966	423.965	−1.963	0.050
Japan					
Constant term	−933.316248	562.194049	154.231	−1.660	0.099
Female sex	1.996612	3.315609	104.626	0.602	0.548
Calendar year	0.488912	0.279931	154.230	1.747	0.083
Switzerland					
Constant term	37.165305	28.161538	39,125.737	1.320	0.187
Female sex	0.130342	0.144640	24,925.454	0.901	0.368
Calendar year	0.002333	0.014026	39,122.296	0.166	0.868
Canada					
Constant term	−113.393911	476.054030	132.791	−0.238	0.812
Female sex	−2.046769	3.297757	73.295	−0.621	0.537
Calendar year	0.077849	0.237227	132.790	0.328	0.743
Liechtenstein					
Constant term	1182.645518	516.645857	102.882	2.289	0.024
Female sex	2.754826	2.269941	72.517	1.214	0.229
Calendar year	−0.569271	0.257412	102.881	−2.212	0.029
United States of America					
Constant term	237.925017	383.185261	297.972	0.621	0.535
Female sex	−1.592948	2.308567	180.960	−0.690	0.491
Calendar year	−0.098054	0.190859	297.985	−0.514	0.608
Belgium					
Constant term	−304.145888	500.299320	132.133	−0.608	0.544
Female sex	1.923155	3.303639	100.875	0.582	0.562
Calendar year	0.172307	0.248996	132.127	0.692	0.490
Spain					
Constant term	519.395530	568.357363	74.188	0.914	0.364
Female sex	−0.202724	2.587542	60.661	−0.078	0.938
Calendar year	−0.237530	0.282985	74.188	−0.839	0.404
Poland					
Constant term	−2195.393095	875.131990	54.937	−2.509	0.015
Female sex	−3.631775	3.857273	56.539	−0.942	0.350
Calendar year	1.113417	0.435743	54.943	2.555	0.013

Data for Non-African runners are sorted in order of the number of finishers of each country

### Performance of the fastest and age of the youngest

Table 12 presents running speed and age of female and male half-marathoners and marathoners sorted from the fastest to the slowest and from the youngest to the oldest. In absolute values, women from Kenya and Ethiopia were running the fastest. Kenyan women were not faster than Ethiopian women (p > 0.05) but they were significantly faster than all other women (p < 0.001 to p < 0.0001). Ethiopian women were not faster than women from Kenya, Portugal, Principality of Liechtenstein and Hungary (p > 0.05), but significantly faster than all other women (p < 0.001 to p < 0.0001). For men, Kenyans and Ethiopians were running the fastest regarding in absolute terms. Kenyan men were not faster than Ethiopian men (p > 0.05), but significantly faster than all other men (p < 0.001 to p < 0.0001). Ethiopian men were not faster than men from Portugal, Principality of Liechtenstein, Italy, Switzerland and Hungary (p > 0.05), but significantly faster than all other men (p < 0.001 to p < 0.0001).

**Table 12:** Running speed and age of half-marathoners and marathoners sorted by country

Running speed				Age			
Country	Women	Country	Men	Country	Women	Country	Men
Half-marathon							
Kenya	14.2 ± 5.1	Kenya	12.7 ± 4.8	Ethiopia	29.8 ± 7.7	Ethiopia	28.0 ± 5.2
Ethiopia	12.8 ± 5.1	Ethiopia	11.1 ± 4.4	Kenya	30.2 ± 6.0	Kenya	29.7 ± 8.3
Portugal	11.4 ± 3.5	Portugal	11.1 ± 2.9	Russia	35.2 ± 9.3	Russia	37.1 ± 9.0
Liechtenstein	10.9 ± 2.5	Liechtenstein	10.5 ± 2.7	Czech Republic	35.6 ± 8.1	Czech Republic	37.5 ± 10.7
Hungary	10.7 ± 2.4	Italy	10.4 ± 3.2	Argentina	38.1 ± 6.9	Poland	38.3 ± 11.3
Italy	10.7 ± 3.1	Switzerland	10.4 ± 2.9	India	38.3 ± 8.9	South Africa	38.7 ± 9.3
Switzerland	10.4 ± 2.9	Hungary	9.9 ± 2.9	Slovenia	38.5 ± 2.1	Canada	38.9 ± 11.9
India	10.2 ± 1.2	France	9.5 ± 3.3	Ireland	38.5 ± 7.5	Australia	38.9 ± 9.9
Spain	10.0 ± 3.0	Netherlands	9.5 ± 3.3	USA	38.5 ± 10.9	Argentina	39.2 ± 6.3
Ireland	9.8 ± 3.0	Australia	9.5 ± 2.9	Great Britain	38.8 ± 9.6	India	39.3 ± 8.6
Argentina	9.6 ± 3.1	Spain	9.4 ± 3.0	Poland	39.1 ± 9.9	Portugal	39.5 ± 9.2
France	9.5 ± 3.3	Norway	9.1 ± 3.0	Canada	39.2 ± 10.0	USA	39.9 ± 10.8
Netherlands	9.5 ± 3.2	Great Britain	9.0 ± 3.2	Greece	39.5 ± 9.3	Greece	39.9 ± 11.2
Russia	9.5 ± 2.8	Israel	8.9 ± 3.3	Denmark	40.4 ± 9.8	Ireland	40.2 ± 9.2
Norway	9.5 ± 2.9	Belgium	8.8 ± 3.0	Spain	40.6 ± 9.2	Spain	40.3 ± 9.6
Great Britain	9.2 ± 3.1	Czech Republic	8.8 ± 3.4	Mexico	40.6 ± 8.5	Great Britain	40.4 ± 10.4
Brazil	9.2 ± 2.5	Ireland	8.7 ± 3.1	Luxembourg	41.0 ± 9.8	Mexico	40.8 ± 9.0
Mexico	9.2 ± 2.4	India	8.7 ± 2.5	Austria	41.1 ± 8.5	Switzerland	41.2 ± 10.3
Czech Republic	9.1 ± 3.6	Mexico	8.7 ± 3.3	Liechtenstein	41.1 ± 9.7	Liechtenstein	41.2 ± 9.2
Greece	8.8 ± 2.7	Greece	8.6 ± 3.1	Switzerland	41.3 ± 10.3	Luxembourg	41.3 ± 9.2
USA	8.7 ± 3.1	Poland	8.5 ± 3.6	France	41.4 ± 9.5	Denmark	41.6 ± 10.7
Denmark	8.6 ± 3.0	USA	8.1 ± 3.0	Belgium	42.0 ± 10.4	France	41.6 ± 9.6
Israel	8.6 ± 3.6	Germany	8.4 ± 3.2	Portugal	42.3 ± 8.7	Slovenia	41.6 ± 16.2
South Africa	8.5 ± 2.7	Argentina	8.4 ± 3.0	Australia	42.3 ± 8.7	Netherlands	41.7 ± 10.6
Poland	8.5 ± 3.5	Russia	8.3 ± 2.7	Italy	42.3 ± 9.6	Belgium	41.8 ± 10.7
Belgium	8.4 ± 3.0	Denmark	8.2 ± 2.9	Israel	42.5 ± 12.8	Finland	42.1 ± 11.7
Germany	8.4 ± 3.2	Sweden	8.1 ± 3.1	Sweden	42.6 ± 11.9	Austria	42.3 ± 9.1
Australia	8.2 ± 2.9	Brazil	8.0 ± 2.9	South Africa	43.3 ± 9.9	Norway	42.5 ± 12.6
Sweden	8.2 ± 3.1	Austria	7.9 ± 3.1	Germany	43.3 ± 9.7	Israel	42.6 ± 11.9
Luxembourg	8.1 ± 2.8	Slovenia	7.9 ± 3.1	Brazil	43.7 ± 10.9	Italy	42.8 ± 9.5
Austria	7.9 ± 3.1	South Africa	7.8 ± 3.2	Netherlands	44.1 ± 9.7	Greece	43.1 ± 9.9
Canada	7.2 ± 3.3	Luxembourg	7.8 ± 2.9	Finland	45.6 ± 10.7	Sweden	43.4 ± 11.8
Slovenia	7.1 ± 2.9	Canada	7.4 ± 3.1	Hungary	48.1 ± 11.5	Hungary	44.2 ± 13.1
Finland	6.6 ± 2.8	Finland	7.0 ± 2.6	Norway	48.3 ± 13.5	Brazil	44.6 ± 9.9
Japan	6.2 ± 2.6	Japan	6.5 ± 2.9	Japan	48.8 ± 14.2	Japan	49.5 ± 15.8
Marathon							
Ethiopia	18.8 ± 0.3	Kenya	17.8 ± 1.3	Ethiopia	26.3 ± 5.5	Ethiopia	27.2 ± 4.6
Kenya	18.3 ± 0.1	Ethiopia	16.1 ± 1.6	Kenya	33.5 ± 2.1	Kenya	29.2 ± 6.0
Liechtenstein	16.6 ± 3.5	Liechtenstein	16.6 ± 3.5	Poland	38.5 ± 11.6	Liechtenstein	40.3 ± 9.0
Italy	15.8 ± 4.1	Switzerland	14.7 ± 4.0	Great Britain	39.0 ± 10.4	Great Britain	40.4 ± 10.2
Switzerland	15.0 ± 4.1	Belgium	14.4 ± 3.9	Canada	40.2 ± 10.0	Poland	40.5 ± 13.1
Japan	14.1 ± 4.4	Spain	14.2 ± 3.9	Liechtenstein	41.2 ± 10.3	USA	41.3 ± 10.6
Spain	13.6 ± 2.8	Italy	13.6 ± 3.9	Austria	41.5 ± 8.5	Canada	41.4 ± 11.1
France	13.4 ± 3.9	France	13.3 ± 3.8	Spain	41.8 ± 7.8	Switzerland	41.8 ± 10.5
Great Britain	13.2 ± 3.9	Great Britain	13.3 ± 3.9	Switzerland	41.9 ± 10.7	Belgium	42.1 ± 11.0
Poland	12.9 ± 3.4	Germany	12.9 ± 3.6	USA	43.3 ± 16.5	Spain	42.3 ± 9.5
Germany	12.9 ± 3.8	USA	12.8 ± 3.9	Belgium	43.4 ± 11.7	Austria	42.9 ± 8.5
Austria	12.4 ± 3.0	Austria	12.3 ± 2.9	France	43.6 ± 9.2	France	43.2 ± 9.7
USA	12.3 ± 3.8	Japan	12.1 ± 4.1	Germany	43.8 ± 10.2	Germany	43.4 ± 9.6
Canada	11.9 ± 4.4	Poland	11.9 ± 3.3	Italy	45.0 ± 12.2	Italy	43.5 ± 9.9
Belgium	11.6 ± 2.6	Canada	11.8 ± 4.3	Japan	51.8 ± 14.9	Japan	48.0 ± 15.5

Considering age, women from Ethiopia and Kenya were the youngest in absolute terms. However, Ethiopian women were not younger than women from Russia, Czech Republic, Argentina, India, Slovenia, Ireland, USA, Great Britain, Poland, Canada, Greece, Denmark and Spain (p > 0.05). Considering athletes from the other countries, women from Ethiopia were significantly younger (p < 0.001 to p < 0.0001). For men, runners from Kenya and Ethiopia were the youngest in absolute values. However, they were not younger than athletes from Russia, Czech Republic, Poland, South Africa, Canada, Australia, Argentina, India, Portugal, USA and Greece (p > 0.05) but significantly younger than men from all other countries (p < 0.001 to p < 0.0001).

In marathon, women from Ethiopia and Kenya were faster than women from all other countries (p < 0.001 to p < 0.0001). However, Ethiopian women were not faster than Kenyan women (p > 0.05). For men, the fastest running speeds were achieved by athletes from Kenya, Ethiopia and Principality of Liechtenstein. Kenyan men were faster than men from all other countries (p < 0.001 to p < 0.0001) with the exception of Ethiopian men (p > 0.05). Ethiopian men were, however, not faster than men from Liechtenstein, Switzerland, Belgium, Spain, Italy, France, Great Britain, Germany and USA (p > 0.05).

Women from Ethiopia and Kenya were the youngest in absolute terms. However, only women from Japan were significantly older than women from Ethiopia (p = 0.001) but not all other women (p > 0.05). Considering Kenyan women, no statistical significant differences were found between the countries (p > 0.05). For men, Ethiopians and Kenyans were the youngest in absolute terms. Ethiopian men were not younger than Kenyan men (p > 0.05), but significantly younger than men from all other countries (p < 0.001 to p < 0.0001). Men from Kenya were not younger than men from Liechtenstein, Great Britain, Poland and the USA, but significantly younger than men from all other countries (p < 0.001 to p < 0.0001).

## Discussion

This study intended to investigate performance and age of female and male Ethiopian and Kenyan half-marathoners and marathoners competing in races held in one country. The most important findings for female and male half-marathons and marathoners from Ethiopia and Kenya were that, (1) they accounted for less than 0.1 %, (2) they were running the fastest and, (3) they were the youngest.

### Low participation of East African runners

A first important finding was that runners from Kenya and Ethiopia accounted for less than 0.1 % in both half-marathons and marathons. The small percentage of participants from these countries should be attributed partially to the distance between these countries and the place of race. Considering the nationality of participants, one might observe a very large number of local participants followed by participants from the neighbouring countries.

Although athletes from neighbouring countries such as Germany, France, Italy and Austria were very numerous, also athletes from very remote countries such as the United States, Japan and Australia competed more numerous than athletes from Ethiopia and Kenya. A very likely explanation could be the income of persons living in these countries since they need to spend money for the travel to and the stay in Switzerland. Costs of living are very high in Switzerland compared to other countries (www.numbeo.com/cost-of-living/country_result.jsp?country=Switzerland). When we compare the gross domestic product (GDP) per capita for persons living in East African countries such as Ethiopia (www.indexmundi.com/ethiopia/gdp_per_capita_%28ppp%29.html) and in Kenya (http://www.indexmundi.com/kenya/gdp_per_capita_%28ppp%29.html) with $1300 and $1800, respectively, persons from the other countries such as the United States of America (www.indexmundi.com/united_states/gdp_per_capita_%28ppp%29.html), Japan (www.indexmundi.com/japan/gdp_per_capita_%28ppp%29.html) and Australia (www.indexmundi.com/australia/gdp_per_capita_%28ppp%29.html) have a GDP of $52,800, $ 37,100, and $43,000, respectively. With these higher GDP, persons from the United States of America, Japan and Australia might easier travel to Switzerland for competing in a marathon than persons from Ethiopia and Kenya.

The finding that mainly local athletes compete in races followed by athletes from surrounding countries confirms recent findings for other races. For example, in long-distance triathletes competing in the ‘Ironman Hawaii’, women and men from the United States of America dominated both participation and performance (Dähler et al. 2014). In solo swimmers crossing the ‘English Channel’ between 1875 and 2013, the most representative nations in the ‘English Channel Swim’ were Great Britain, the United States of America, Australia and Ireland. The fastest swim times were, however, not achieved by local athletes but by athletes from the United States of America, Australia and Great Britain (Knechtle et al. 2014).

However, the most likely explanation for the very low participation of East African runners in half-marathons and marathons held in Switzerland are economic reasons. For Kenyan runners, marathon running is a means of making money to help their families, parents and siblings (Onywera et al. 2006; Onywera 2009). Onywera (2009) described economic reasons for Kenyan athletes as one of the most important factors to compete in marathon running, which might be undercharged so far (Hamilton and Weston 2000). Prize money in Swiss half-marathons and marathons is very low compared to prize money offered in the ‘World Marathon Majors’ (www.worldmarathonmajors.com). For the winner in the ‘Zurich Marathon’ in Switzerland, the prize money is 10,000 Swiss Francs (www.zurichmarathon.ch) which is very low in contrast to the prize money offered in large city marathons. Indeed, overall prize money in races of the ‘World Marathon Majors’ is considerably higher (www.worldmarathonmajors.com). In the ‘BMW Berlin Marathon‘, the ‘Tokyo Marathon’, and the ‘Virgin London Marathon’ the prize money is $1,000,000, in the ‘Boston Marathon’ $846,000, in the ‘TCS NYC Marathon’ $805,000 and in the ‘Bank of America Chicago Marathon’ $560,000 (www.bestroadraces.com/brr100.php/prizes). The differences in prize money seem very similar in half-marathon compared to marathon. In a large half-marathon held in Switzerland such as the ‘Hallwilerseelauf’, the prize money for both women and men for the top five is, however, only CHF 600, 400, 300, 200, and 100, respectively (www.hallwilerseelauf.ch). In an elite half-marathon such as the **‘**IAAF/AL-Bank World Half Marathon Championships’, a total prize purse of US$245,000 will be paid by the IAAF for the men’s and women’s races (www.iaaf.org/news/news/prize-money).

### East African runners were the fastest in half-marathons and marathons

A second finding was that female and male runners from Kenya and Ethiopia were the fastest in both half-marathons and marathons. The dominance of East African runners was evident for both marathon and half-marathon but differed from longer distances. For instance, it has been shown that male Japanese runners were the best in 100-km ultra-marathons (Cejka et al. 2014). The trend in performance across years should be explained by a model showing that human speed after having progressed fast in the past has now reached a plateau and further progression should be attributed to an enlarged population of runners and improved training practices (Desgorces et al. 2012).

### East African runners were the youngest in half-marathons and marathons

A third important finding was that women and men from Kenya and Ethiopia were the youngest in both half-marathons and marathons. Their mean age is considerably lower as has been reported for elite and recreational marathoners. The age of elite marathoners is at around 29–30 years when the nationality was not considered (Hunter et al. 2011). In female and male marathoners competing between 1979 and 2014 in the ‘Stockholm Marathon’, the age of the fastest marathon performance was even higher with 34.3 ± 2.6 years (Lehto 2015). In a study investigating runners competing in Swiss half-marathons and marathons from 2000 to 2010 and considering the top five African and Non-African runners, the mean age of the male runners was significantly higher for Non-African runners than for African runners in both half-marathons (Non-African runners 31.1 ± 6.4 years, African runners 26.2 ± 4.9 years) and marathons (Non-African runners 33.0 ± 4.8 years, African runners 28.6 ± 3.8 years). In marathons, the top five female Non-African runners (31.6 ± 4.8 years) were ~4 years older than the top five female African runners (27.8 ± 5.3 years) (Aschmann et al. 2013). The difference in age between East Africans and Europeans found in the present study was not in agreement with a previous comparison between African and non-African runners of marathons and half-marathons (Cribari et al. 2013) indicating that the younger age was a specific characteristic of East Africans and should not be generalized to all African runners.

### Physiological interpretation

For the dominance of East African runners such as Kenyan runners, physiological aspects need to be considered (Larsen 2003; Larsen and Sheel 2015). It has been supported that running speed sustained over a prolonged time depends on the maximal sustainable VO_2_ (oxygen uptake) and running economy (Millet et al. 2012). A comparison between European and Eritrean long-distance runners showed that Eritreans, despite having a lower VO_2_max (maximum oxygen uptake), had a better running economy at 19 km h^−1^ (Santos-Concejero et al. 2015). A better running economy might explain the supremacy of East Africans in the marathon, and the delayed glycogen depletion and reduced thermal stress have been suggested to be associated with a better running economy (Millet et al. 2012). An exceptional biomechanical and metabolic economy, chronic exposition to altitude, sociocultural background and a strong psychological motivation were highlighted as other factors of this supremacy (Onywera 2009; Wilber and Pitsiladis 2012). Moreover, the impact of stereotypes has also been noticed because, independently from the possible existence of physiological advantages in East Africans, the belief that such differences exist can impact performance by creating a psychological atmosphere (Baker and Norton 2003). With regards to their nutritional habits, a research on the dietary intake of Ethiopian long distance runners has shown that they met most recommendations for endurance athletes (Beis et al. 2011). A study on the diet of Kenyan endurance runners revealed that it composed mostly by carbohydrates (~67 %) and less by protein (~15 %) or fat (~17 %) (Fudge et al. 2006).

In addition to the abovementioned physiological factors, Eastern African runners might differ from runners of other origin with regards to other specific anthropometric characteristics (Kohn et al. 2007; Lucia et al. 2006; Prommer et al. 2010; Vernillo et al. 2013). For instance, compared to elite German 10-km runners, elite Kenyan runners had a similar VO_2_max (ml min^−1^ kg^−1^) but were lighter by more than 9 kg (Prommer et al. 2010). Xhosa 10-km runners had also similar VO_2_max (ml min^−1^ kg^−1^) as their Caucasian counterparts, but they were lighter and shorter (Kohn et al. 2007). Eritrean distance runners had a lower body mass index and a better running economy at 21 km h^−1^ than Spanish runners, whereas their VO_2_max was similar (Lucia et al. 2006). In top class Kenyan marathoners, ectomorphy is dominant, but endomorphy and mesomorphy is more than one-half unit lower (Vernillo et al. 2013).

A review of genetic and lifestyle factors of the performance of the East Africans distance runners concluded that the findings on candidate genes linked to performance of Caucasian populations were not confirmed in East Africans showing research methods’ limitations and the polygenic nature of performance (Tucker et al. 2013). This was in agreement with another review showing that distance running success of East Africans was not based on a unique genetic profile (Wilber and Pitsiladis 2012). Another parameter that has not been studied previously as much as the abovementioned parameters might be the physical activity and inactivity levels when athletes did not practise their sport. Surprisingly, a study in marathon and half-marathon runners showed that these athletes trained for 6.5 h weekly, but they also spent much more time sitting (Whitfield et al. 2014). The aforementioned study found no relationship between sitting time and performance. However, potential differences in non-sport physical activities and inactivity levels between East Africans and Europeans should be examined in future studies.

### Limitations

A limitation of this analysis is the fact that an athlete may have changed his/her nationality, where, for example, an athlete from an African country might have been naturalized in another country. As an example, the Swiss marathoner Tadesse Abraham was born in Eritrea but is now a Swiss citizen. He won three marathons and one half-marathon in Switzerland (www.tadesse-abraham.ch). On the other hand, the focus of the present study was on half-marathon runners’ characteristics (i.e. age, participation and performance) with regards to marathon. Since there was no evidence that the above-mentioned concern about the nationality appeared differently to the two events (half-marathon vs. marathon), it might be supported that it did not affect the overall findings.

## Conclusions

In summary, women and men from Kenya and Ethiopia, despite they accounted for less than 0.1 % in half-marathons and marathons, achieved the fastest race times and were the youngest in both half-marathons and marathon. These findings confirmed in the case of half-marathon the trend previously observed in marathon races for a better performance and a younger age in East African runners compared to Non-African runners.
